# Photosynthetic Diel Cycling Influences Inverse Zinc and Copper Solubility Dynamics within a Constructed Wetland

**DOI:** 10.1021/acs.est.5c12039

**Published:** 2026-04-02

**Authors:** Zhaoxun Yang, Gary F. Vanzin, Michael A. P. Vega, Adam R. Brady, Lino Morales Paredes, James F. Ranville, Jonathan O. Sharp

**Affiliations:** † Department of Civil and Environmental Engineering, 3557 Colorado School of Mines, Golden, Colorado 80401, United States; ‡ Center for Mining Sustainability, Colorado School of Mines, Golden, Colorado 80401, United States; § Departamento Académico de Química, Facultad de Ciencias Naturales y Formales, 121568 Universidad Nacional de San Agustín de Arequipa, Arequipa 04001, Peru; ∥ Department of Chemistry, Colorado School of Mines, Golden, Colorado 80401, United States; ⊥ Hydrologic Science and Engineering Program, Colorado School of Mines, Golden, Colorado 80401, United States

**Keywords:** metal, diel cycle, wetland, photosynthesis, algae

## Abstract

Shallow unit process open water (UPOW) wetlands host a photosynthetic microbial biomat rather than emergent plants. Sunlight-associated maxima during diel cycles in water column pH (∼7–10) and dissolved oxygen (∼5–20 mg/L) within this nature-based construct have implications for biogeochemical processes and contaminant attenuation. In field applications, an inverse solubility dynamic was observed with decreases in zinc and increases in copper, in association with photosynthesis. Laboratory flow-through photosynthetic bioreactors amended with field-derived biomat reproduced key geochemical diel variables and harbored similar microbial structure and function. Sequential extractions of solid phases within flow-through bioreactors and isotope-enriched microcosms revealed that Zn and Cu primarily accumulated in the chemically labile phases of the surficial water–biomat interface layer. Equilibrium modeling suggested that diel changes in Zn were influenced by calcite sorption/coprecipitation. However, rather than analogous precipitation at higher pH, Cu experienced prominent influences from organic associations with sediments and dissolved organic carbon ligands. Retrospective analysis of diel cycling in other published field and laboratory studies indicates a potential concentration-related diel cycling pattern for Cu but not for Zn. These findings collectively inform a more mechanistic understanding of photosynthetic influences on diel aqueous geochemical metal cycling in analogous systems such as wetlands and periphyton-influenced streams.

## Graphical abstract

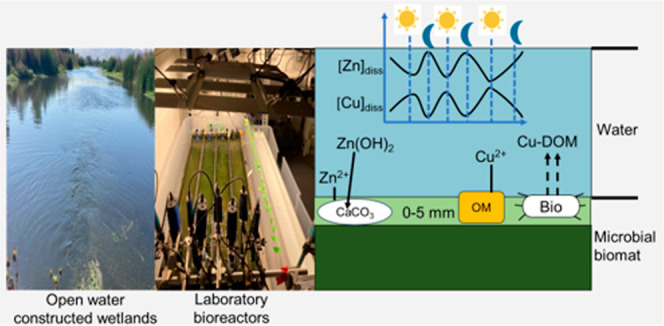

## Introduction

Diel cycles are widely observed in streams,^1–6^ rivers,^7–9^ coastal waters,^10^ and natural and constructed wetlands.^11–13^ For surface water systems that are influenced by photosynthesis, the consumption of inorganic carbon dominates during the day versus its introduction via respiration at night. This is closely linked with diel cycling of pH, dissolved oxygen (DO), and other geochemical variables with implications for the temporally dynamic behavior of metals.^14–16^ Mechanisms for metal removal by constructed wetlands include a variety of biological and abiotic reactions such as precipitation, interactions with solid phases (e.g., adsorption), aqueous ligands (e.g., complexation), plant uptake, and microbial transformations.^14,16^ While these processes exert recognized influences on metal solubility, their coupled yet perplexing behaviors under photosynthetic conditions with pronounced diel shifts of pH and DO in constructed wetlands have not been previously demonstrated. This is particularly important for understanding the mobility of ecotoxicologically relevant elements such as zinc (Zn) and copper (Cu) where speciation affects their toxicity.^17^

Unlike more traditional surface flow vegetated wetlands, shallow unit process open water (UPOW) constructed wetlands are designed with a benthic geotextile liner that limits the establishment of roots for emergent plant growth.^18^ Due to this design, UPOW wetlands are colonized by a photosynthetic benthic sedimentary layer (biomat) comprised of minerals, algae, and bacteria. This results in intertwined metabolic processes where photosynthesis dominates during the day and heterotrophic respiration dominates during the night, which in turn exerts prominent geochemical influences on diel water column chemistry.^19–21^ The surficial layer of the biomat is biogeochemically dynamic and can directly react with and influence water column metal concentrations. Thus, information for both abiotic (e.g., mineral phases) and biotic (microbial community compositions) components of the biomat is needed. The stability of biomat-associated metals may experience further changes due to temporal accretion associated with microbial and algal growth and senescence.

Demonstration-scale UPOW treatment cells at the Prado Constructed Wetlands Complex in Corona, CA receive water from the Santa Ana River year-round, which is dominated by tertiary-treated municipal wastewater effluent during summer base flows.^19^ Past work at this site has revealed mechanisms associated with the attenuation of multiple contaminant classes including nutrients,^21–23^ pesticides,^24^ and pharmaceuticals,^21,25,26^ as well as capacity for metal attenuation.^27^ The latter study revealed that Zn, Cu, Pb, and Ni were removed through a combination of assimilative and sorptive reactions; however, the influence of diel shifts on the overall metal fate and transport in this system is not well-understood. Past work has demonstrated that field-relevant diel cycles can be mimicked in the laboratory using LED-illuminated bioreactors containing a field-derived biomat,^28–30^ enabling more controlled mechanistic investigations into their influence on metal fate and transport.^1,6^

We hypothesized that diel changes in water column geochemistry interact dynamically with metal sequestration at the surficial layer of the biomat and act as primary regulators of metal attenuation in UPOW wetlands. Preliminary field observations suggested a diel cycle where the dissolved Cu concentration increases during the day and decreases at night.^12^ This is a paradoxical pattern that is the inverse of dissolved zinc; however, similar behavior has been observed in mountain rivers with limited mechanistic understanding.^7^ Based on discussions in the previous literature that reported Zn and Cu diel cycling, we hypothesized that in our system, diel changes of dissolved Zn were affected by calcite precipitation and those of dissolved Cu were affected by dissolved organic matter. To query this, we confirmed the function similarities between field-scale constructed UPOW wetlands and flow-through photosynthetic laboratory bioreactors containing field-derived biomats through both geochemical comparisons of metal removal rate constants and ecological comparisons of microbial communities. Depth-resolved cores from the bioreactors enabled the tracking of changes in metal concentrations in solid phases at the biomat surficial layer. Batch microcosms were further enriched in stable ^65^Cu and ^68^Zn isotopes. This stable isotope amendment enabled quantification of metal sequestration by the surficial biomat phases. Based on experimental results and equilibrium geochemical modeling, we propose a conceptual model for the intertwined geochemical influences on Cu and Zn dynamics. Finally, we contextualized the observed metal dynamics by comparing them to other natural and engineered systems that have documented analogous diel cycles to better infer when identified drivers are most likely to exert their influence.

## Materials and Methods

### UPOW Laboratory Bioreactors and Field Systems

An existing photosynthetic bioreactor design^29,30^ was modified to include four parallel bioreactors seeded with Prado-derived UPOW wetland biomats^21^ and one biomat-free control (Figure S1, Table S1). The influent was delivered using a multichannel Ismatec IPC pump drawing from five media containers, maintaining a system hydraulic residence time of 17–24 h (Table S1) and a diel cycle of 11 h light (353–706 mmol/s/m^2^ photosynthetic photon flux density)^29^ and 13 h dark. The influent medium was a variation of a diatom growth recipe^31^ that reduced the silica concentrations by a factor of 100 and boron and phosphorus concentrations by a factor of 10 (Table S2). After an 8 day conditioning phase with a lower metal addition of ∼40 μg/L Zn and ∼20 μg/L Cu, the influent medium was amended with ∼200 μg/L Zn and ∼100 μg/L Cu (Figure S2) and 0.25 mg/L NaBr as a conservative tracer. Dissolved oxygen (Hach HQ40d, LDO101) and pH (Vernier Go Direct) were monitored at 30 min intervals. Water samples were collected from the overlaying water column near the bioreactor outflow (“Outlet”) and the inflow tubes (“Inlet”). Field-based water column samples were collected in 2018 from a demonstration-scale UPOW treatment cell at the Prado Constructed Wetlands Complex (Figure S5).^12,21,32^

Light and dark areal rate constants (meter per year) for metal removal in the bioreactors were calculated based on a tanks-in-series model and compared to field observations (details in Supporting Information Section S2.1).^23,33,34^ Batch microcosms using the same media were established to evaluate the role of dissolved organic carbon in Cu and Zn removals (details in Supporting Information Section S3).

### Depth-Resolved Geochemical and Microbial Analysis of the Biomat

Duplicate biomat cores (∼1 cm diameter) were harvested from flow-through bioreactors at the beginning and end of the experiments (Figure S2a) using a modified syringe as a coring device.^21^ The cores were flash-frozen in liquid nitrogen and stored at −80 °C. Cores were sectioned into sterile 5 mm depth segments (0–5 mm, 10–15 mm, and 20–25 mm) using a razor blade and further divided into three units for microbial community analysis, geochemical sequential extractions, and sample archives at −80 °C, as outlined in Figure S3 and prior studies.^21^ The biomat for microbial community analysis was preserved in 750 μL DNA/RNA shield (Zymo Research, Inc.), while portions for sequential metal extracts were transferred to a 15 mL tube.

A modified sequential extraction protocol (Table S3) was applied to explore how experimental conditions influence the association of metals with the biomat. Four phases were operationally defined as water-extractable; adsorbed/carbonates/exchangeable + labile organics (ACE + LO) extracted by 0.1 M sodium pyrophosphate; oxidizable extracted by 30% hydrogen peroxide and 3.2 M ammonium acetate; and acid-extractable by 1% nitric and hydrochloric acid.^35,36^ Due to the nature of sodium pyrophosphate and no strong preceding reagents, the ACE + LO phase would include the metals adsorbed to the minerals as well as complexed with humic and fulvic substances.^37^ Full details are provided in Supporting Information Section S4.

Data for microbial community characterization in each depth section were generated by amplicon sequencing for 16S/18S rRNA genes with 515F_Y and 926R primers as described previously.^21,26,38^ Demultiplexed 16*S*/18S rRNA sequencing forward and reverse reads of field cores from 2018 (*n* = 40) were downloaded from NCBI BioProject PRJNA818364. All samples used in the analysis are listed in Table S9 (for Figures 2 and S6–S12) and under BioProject PRJNA1414098. DNA extraction, additional sequencing steps, and data processing details are provided in Supporting Information Section S3. The final samples were delivered to the University of Colorado Anschutz Medical Campus Cancer Center Genomics Shared Resource Core Facility (RRID:SCR_021984) and sequenced on an Illumina MiSeq platform using a v2 2 × 250 bp reagent kit.

**Figure 1:**
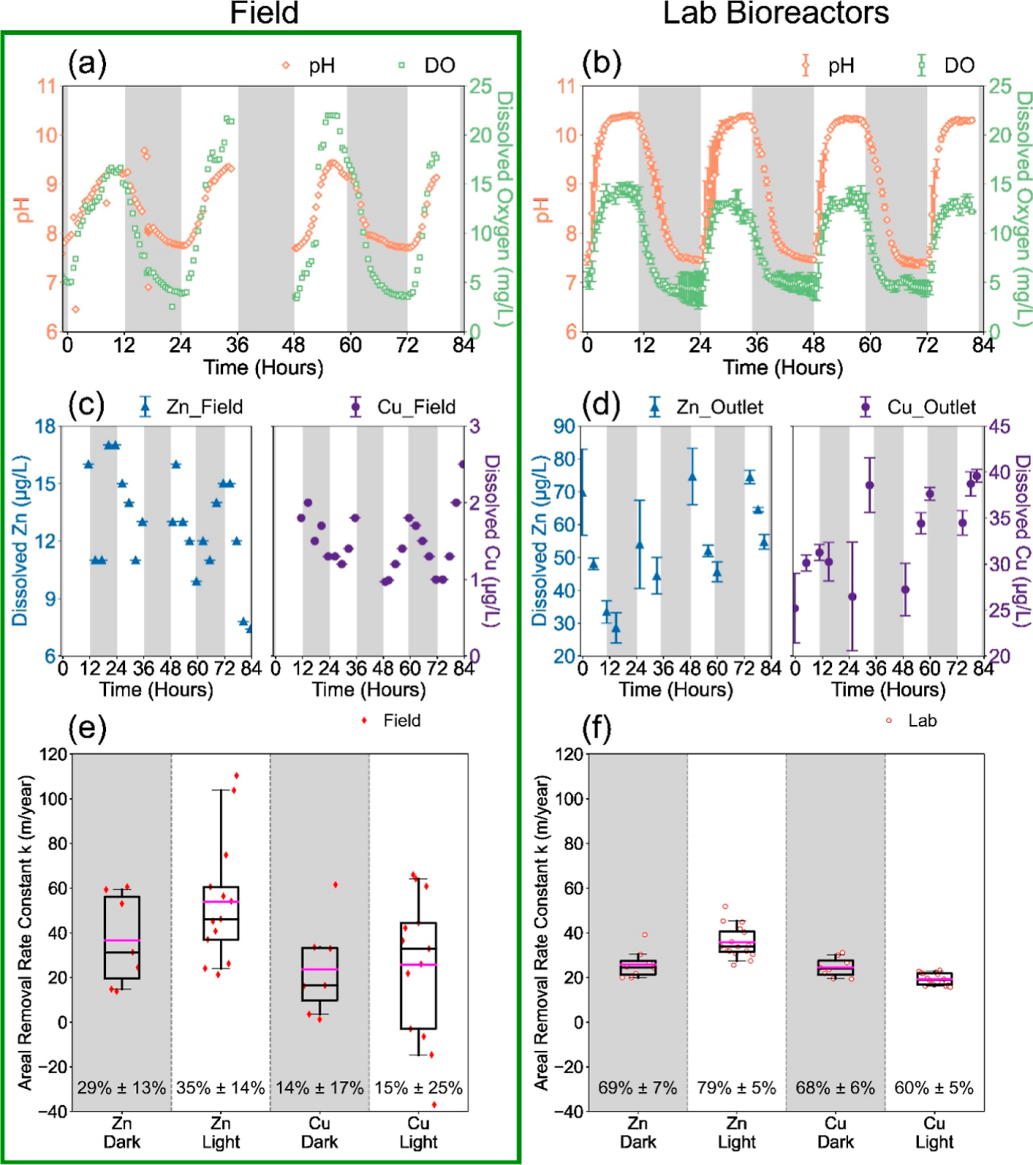
Diel changes in pH (a/b, left *y*-axis) and dissolved oxygen (a/b, right *y*-axis); dissolved Zn and Cu; and areal removal rate constants (calculated by eq S1) for zinc and copper under dark and light conditions in a field-scale UPOW wetland (a,c,e) and laboratory flow-through bioreactors (b,d,f). The error bars in (c) represent standard deviations of measured sample concentrations (*n* = 22). The error bars in (d) represent the ranges of biological duplicates (*n* = 12, as detailed in Figure S2). The bottom numbers in (e,f) show the percent removal calculated from (1 – *C*_out_/*C*_in_) × 100%, with red points indicating the calculated rates for each time point sample. The magenta line indicates the average value of all samples in that category, and the black line indicates the median value. Data for the field-scale UPOW wetland were obtained from field samplings in 2018.

**Figure 2:**
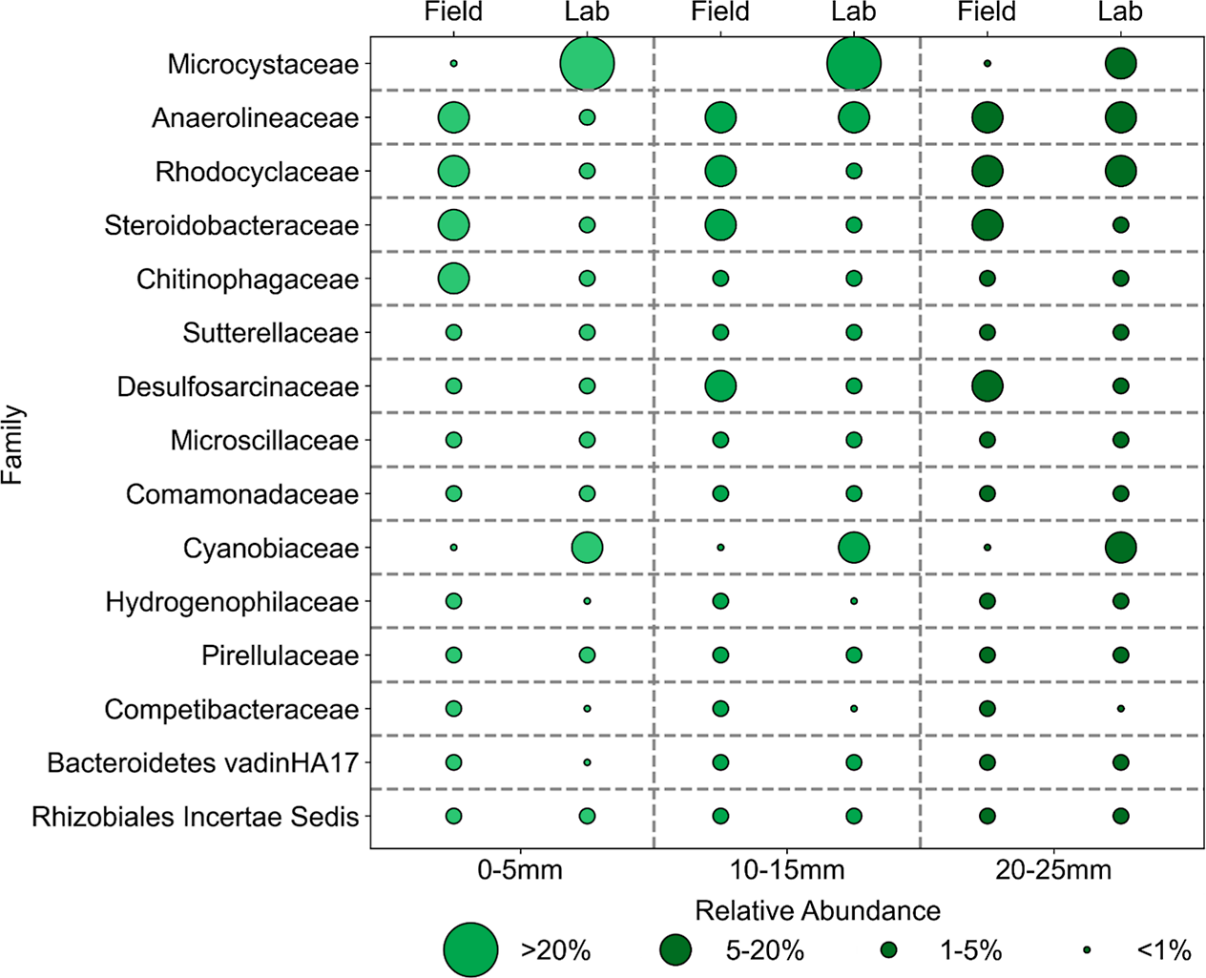
Microbial community composition with top 15 taxa for three depth sections in field (*n* = 65) and lab bioreactors (*n* = 43) by 16S rRNA gene sequencing. Marker sizes indicate the percentages of relative abundance that are the average values of all samples in that category, and the *y*-axis denotes the family level taxonomy. More details on the metadata and bioinformatics are described in Supporting Information Section S2.2.

### Stable Isotope Amendment to Query Metal Partitioning and Fate

Stable isotopes (^68^Zn and ^65^Cu) were used to quantify the accumulation of metals from the water column to the biomat. Batch microcosms in serum bottles contained a 10 mm biomat layer (∼2 g wet biomat), each with ∼50 mL of filtered bioreactor outlet water, either amended with the natural abundance of Zn and Cu or completely ^68^Zn and ^65^Cu (a final mixture of 16.6 ± 1.1 μg dissolved ^68^Zn and 8.8 ± 0.6 μg dissolved ^65^Cu). Full details on the batch microcosm setup, stable isotope amendments, analytical measurements of Zn/Cu isotopes, and subsequent calculations are presented in Supporting Information Section S5. The following equation was used for the calculation of isotopic ratio enrichment

ratioenrichment=(Zn68/Zn66)exp(Zn68/Zn66)nator(Cu65/Cu63)exp(Cu65/Cu63)nat 1

where “exp” is the isotopic ratio in each extraction phase of the biomat in the microcosms at the end of the experiments and “nat” is the natural abundance of the corresponding isotopic ratio.^39^

### Analytical and Computational Approaches

For the analysis of dissolved element concentrations, samples were filtered through a 0.2 μm polyethersulfone (PES) filter, acidified, and diluted as necessary. To determine total element concentrations, samples were preserved in the same manner, except filtration was omitted. Key elements of interest (Zn, Cu, Pb, Cd, Ni, Mn, and Br tracer) were analyzed by Inductively Coupled Plasma Mass Spectrometry (PerkinElmer NexION 350D ICP–MS). Additional elements (Al, Ca, Mg, Na, K, Fe, P, S, and Si) were analyzed by Inductively Coupled Plasma Atomic Emission Spectroscopy (PerkinElmer Optima 8300 ICP-AES). Measurements of major anions, total and dissolved organic carbon (TOC, DOC), DOC characterization by excitation–emission matrix (EEM) fluorescence spectroscopy, specific ultraviolet absorbance (SUVA_254_), humification index,^40^ biological index,^41^ and fluorescence index^42^ are described in Supporting Information Section S3.

PHREEQC^43^ geochemical modeling was applied to explore the speciation of Cu and Zn during diel changes using an extension of a previously established model (Supporting Information Section S6).^27^ Differences and bootstrapping of biomat depth profile comparisons (Figure 3, Supporting Information Section S4) were conducted in R using the dabestr package.^44^ All statistical analyses were performed in MATLAB ver. R2020b,^45^ unless specified otherwise. A p-value <0.05 was considered significant throughout the study.

**Figure 3:**
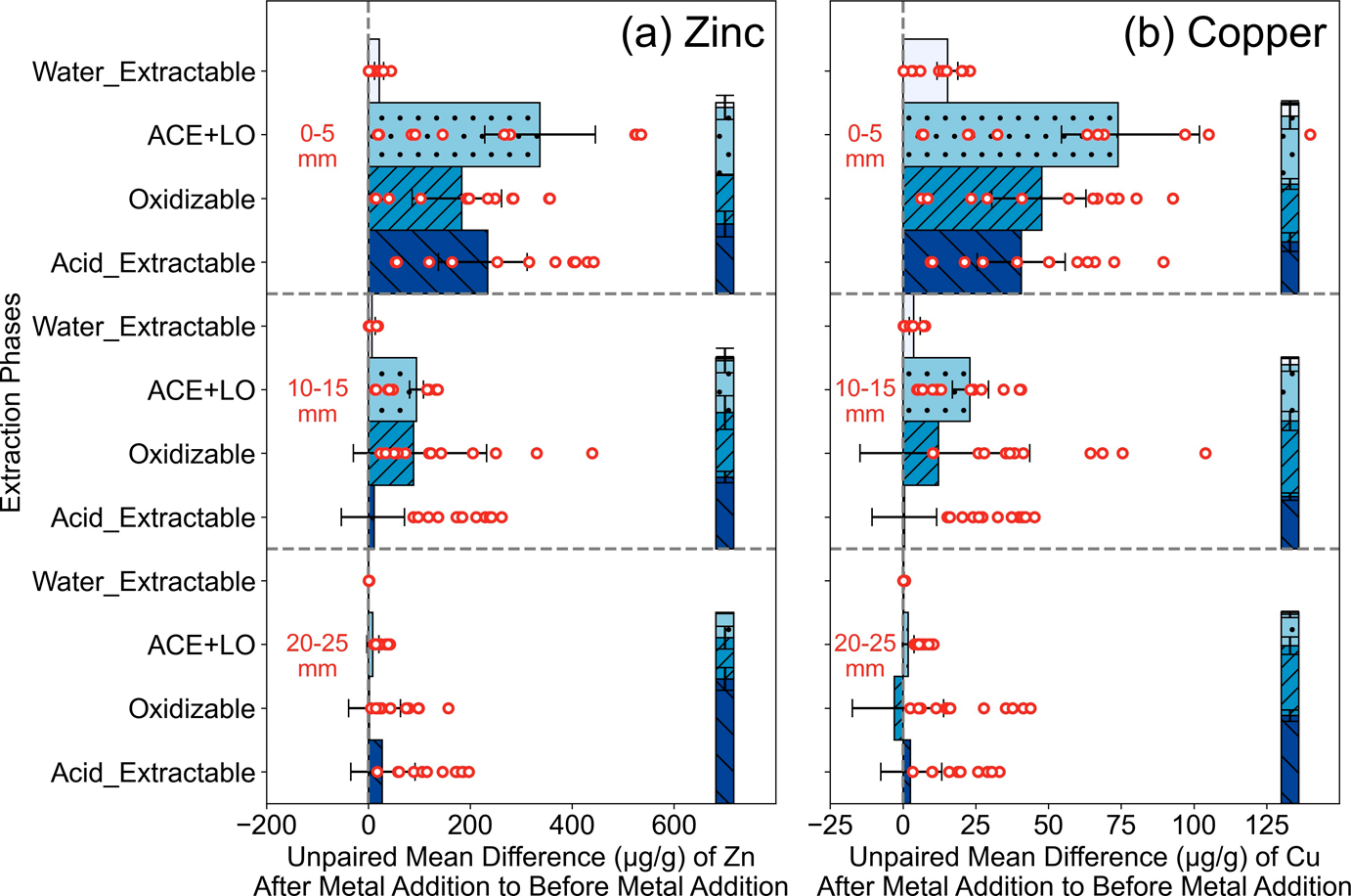
Depth speciation profiles of Zn (a) and Cu (b). Each bar shows the unpaired differences in the flow-through bioreactors after metal addition compared to before metal addition for each sequential extraction phase (indicated by colors and hatches)—water-extractable, adsorbed/carbonates/exchangeable + labile organics (A/C/E + LO), oxidizable, and acid-extractable—at each depth (0–5 mm, 10–15 mm, 20–25 mm). The red points represent the raw data included in the analysis (*n* = 12 for each row). The error bars are 95% confidence intervals after 5000 bootstrapping. The three bars on the right represent the relative percentages of Zn (a) and Cu (b) in each sequential extraction phase (shown as the height of each colored block) at each depth, and error bars show the ranges of duplicate cores.

## Results and Discussion

### Analogous Diel Behavior across Bioreactors and Field Systems

Laboratory flow-through bioreactors exhibited diel pH and DO cycles that peaked in association with light and were analogous to field observations (Figure 1a,b). Analogous diel pH and DO oscillations are widely observed in surface waters where photosynthesis leads to changes in dissolved carbon dioxide and oxygen concentrations that diverge from atmospheric equilibrium.^5,8,28^ Different absolute maxima and delta (max–min) values of pH and DO between field and laboratory bioreactors can be attributed to variables such as differences in water constituents and light intensity.^29^ Diel temperature fluctuations within the laboratory bioreactors were approximately 5 °C, which mimicked but were lower than the ∼15 °C variations observed in field-scale UPOW wetlands in September when the field sampling was conducted.^12^ Although temperature variations can affect oxygen solubility in water, Zn adsorption, and, to a lesser extent, Cu adsorption,^2,7^ the much larger pH and DO shifts and relatively small temperature variations in the laboratory bioreactors suggest that temperature-induced effects are less critical for Zn and Cu diel changes in the laboratory bioreactors.

In both laboratory bioreactors and field wetlands, dissolved Zn concentrations in the water column near the outlet were the highest at the end of dark cycles and decreased over the course of the light cycle. In contrast, dissolved Cu concentrations exhibited an inverse pattern with a lower magnitude of variations (Figure 1c,d). In addition to dissolved Zn and Cu, diel variations in dissolved Ca, Mg, and Mn were also observed (Figures S4, and S5, Table S6), with analogous behavior between the laboratory bioreactors and the Prado constructed wetland complex.^12^

As the laboratory bioreactors exhibited diel geochemical dynamics analogous to those of the field-scale system, we furthered this comparison to include areal metal removal rate constants (meter per year) and percent removal across the systems (Figure 1e,f). The field water inlet concentrations were ∼10-fold lower for Zn and ∼100-fold lower for Cu than the inlet concentrations used in the bioreactor experiments.^12^ Differences in these metal concentrations as well as area and flow rates (Table S8) resulted in different percentages of metal removal between field and laboratory bioreactors, although the patterns were similar. Significant differences (Mann–Whitney U test *p* = 0.003) between dark and light conditions were observed for Zn/Cu removal rate constants in the laboratory bioreactors, but not in the field. Beyond influent concentrations, additional explanations include the more controlled operational conditions, the lack of wind mixing, and more uniform flow when contrasting laboratory flow-through reactors with a field system. However, flow in both lab and field systems was best represented as tanks-in-series models^25,33^ with similar average areal removal rate constants (Figure 1e,f, Mann–Whitney U test, *p* = 0.279), suggesting that field and laboratory bioreactors likely share similar removal mechanisms. This similarity, which is not entirely dependent on influent concentrations, also suggests that the overall removal is a combination of different removal mechanisms involving both zero-order (not concentration-dependent) and first-order (concentration-dependent) reactions.^34^

### Biological Similarities across Bioreactors and Field Systems

To better understand biotic influences on the systems and evaluate the effects of biomat incubation in the laboratory bioreactors versus in the field wetlands, we compared the microbial and eukaryotic community profiles using 16*S*/18S rRNA sequences. Some taxonomic differences were observed (Figure S6) in the comparisons between the field and laboratory bioreactors calculated from Bray–Curtis distance (*R* ^2^ = 0.249, *p* = 0.001, Supporting Information Section S2.2). Members of the Family Microcystaceae were significantly enriched in the bioreactors compared to those in the field (Figures S7 and S8), with particular prominence for the genus *Synechocystis PCC 6803*. The genus *Microcystis PCC-7914*, which is associated with harmful algal blooms,^46,47^ was also observed in the bioreactor but with much lower abundance compared to other cyanobacteria (Figure S8). For 18S rRNA gene sequence communities, an increase of eukaryotic green algae (i.e., Family Sphaeropleales_fa) in the lab compared to that in the field was observed (Figures S10, and S11), which is consistent with previous studies.^21^ A possible explanation is lower silica concentrations during our laboratory operation (∼2 mg/L Si for lab versus ∼12 mg/L Si in the field), which would skew toward non-diatom type algal species instead of the more diatom-dominant environment observed in the field.^19,48^ The microbial communities were generally similar across depths from 0 to 25 mm (Figure 2, *R* ^2^ = 0.02, *p* = 0.339); similar results were observed regardless of the normalization methods used (Supporting Information Section S2.2, Figure S6). No significant differences were observed in taxa when evaluated at log2 fold changes across different depths.

Despite these taxonomic differences, we observed putative functional redundancy of phototrophs^21,49–53^ associated with different family members of the microbial community in both field wetlands and laboratory bioreactors (Figure S9). This could explain why general similarities existed in the geochemical behaviors of the field and lab-based systems despite some taxonomic differences in the microbial community composition, with photosynthesis-driven pH changes governing Zn and Cu attenuation processes when concentrations were below those of pronounced ecotoxicological effects.^54,55^ Both Zn and Cu are involved in the photosynthetic electron transport chain of *Synechocystis PCC 6803* and other phototrophs.^56,57^

Although the inlet concentrations of the laboratory bioreactors were ∼10-fold higher for Zn and ∼100-fold higher for Cu compared to the inlet concentrations in field wetlands, metal addition did not exert a prominent effect on the microbial community composition in the laboratory bioreactors given the variation (*R* ^2^ = 0.089, *p* = 0.005, Figure S6g), with only one significant decrease (Log_2_FoldChange = −7.2, *p* < 0.001) in genus *Sphingopyxis* in the bioreactor samples after metal addition. The flow-through bioreactors were initially seeded with a homogenized field-derived biomat, and the experimental duration of metal addition in the flow-through bioreactors of approximately 2 weeks is short compared to the environmental operation, which might have skewed the contrasts. In other wetlands with long-term metal exposure, significant changes in microbial community composition have been documented.^55,58^ However, the collective evidence presented above regarding pH, DO, temperature, diel shifts in dissolved Zn, Cu, Ca, Mg, and Mn, areal metal removal rate constants, and microbial community composition indicates that the laboratory bioreactors are functionally similar to the field-scale wetlands.

### Inverse Zinc and Copper Solubility during Diel Cycles

With lab-scale bioreactors generally analogous to the field but with the capacity for more experimental control and access, we utilized them to further investigate potential drivers responsible for the inverse responses of dissolved Zn and Cu. The magnitudes of the dissolved Zn diel variations were 2–7 times higher than for Cu, a pattern that has been previously documented in other natural systems including rivers and creeks, independent of when (e.g., sunrise or sunset) the maximum was observed in diel variations.^17,28,59^ Analysis of dissolved versus total (unfiltered) Zn and Cu revealed a dominance of the soluble phase and no discernible shifts in ratios as a function of diel cycling (Table S4).

Bromide as a conservative tracer exhibited comparatively stable behavior throughout the experiments (changes 1–11%, Table S4). Similar stability was observed for aqueous Zn and Cu concentrations (4–17% and 4–9%, respectively) in a bioreactor without a biomat at a constant pH of approximately 7.5. Since the evaporation rates of analogous flow-through bioreactors were estimated to be around 10% of the total flow,^33^ this suggests that the more pronounced diel changes of dissolved Zn (average changes of 101%) and Cu (average changes of 45%) in the presence of the biomat were not induced by systematic experimental conditions such as hydrological constraints or evaporation.

Abiotic and indirect biological factors contributing to pH-induced diel changes in soluble Zn include hydroxide/carbonate precipitation, dissolution/coprecipitation with actively formed calcite and iron/manganese minerals, and adsorption/desorption onto these minerals.^8,60–63^ Previous work^27^ on the mineral composition of the biomat confirmed the presence of iron (oxyhydr)­oxides and calcite. The variations (<5.6 μg/L) and total concentrations (<11.2 μg/L) of both Fe and Mn in the water column were minor compared to diel Zn fluctuations (6.5–58.5 μg/L), suggesting limited impact of diel activity on the formation of Fe/Mn hydroxides that could sorb Zn. However, pH-induced Zn sorption to existing solid-phase iron minerals in the biomat could occur. As for calcite, we observed an increase in calcium in the water column in biomat bioreactors (2.3–4.3 mg/L, Table S6) with diel variations (average changes of 54%, Figure S4g), compared with stable calcium concentrations (1 mg/L, 1–5% changes, Table S6) in the no-biomat bioreactor. This suggests a potential release and exchange of calcium between the water column and calcite minerals in the biomat. Saturation indices of calcite in the PHREEQC model (Table S17) as elaborated later support this mechanism. Calculations based on charge balance of bicarbonate and carbonate with other constituents in solution (Supporting Information Section S3) showed that alkalinity at the end of the light period (23.2–27.5 mg/L as CaCO_3_) were lower than at the beginning (42.7–43.6 mg/L as CaCO_3_, Table S7). This mimicked diel changes of dissolved calcium concentrations and hence is consistent with diel dissolution and precipitation of calcium carbonate and/or biological consumption of CO_2_ and bicarbonate during photosynthesis. Hence, we propose that sorption to iron minerals and sorption/coprecipitation with calcium carbonates both could contribute to diel changes of dissolved Zn.

In contrast, though sorption to iron minerals could also affect dissolved Cu, copper ions have less affinity to calcium carbonate minerals than Zn.^64,65^ There is a literature precedent for linking Cu diel variation patterns to changes in Cu-DOC complexes and Cu-binding extracellular polymeric substances (EPS).^5,10,28,66^ Consistent with this mechanism, we observed higher TOC/DOC concentrations in the water column at the end of the light cycle (2.0 ± 0.3 mg C/L) when contrasted with that at the end of the dark cycle (1.4 ± 0.2 mg C/L, Table S7), which could be attributed to organic exudates released during photosynthesis. This difference was higher than that in the no-biomat bioreactor (0.7 mg C/L at the end of the light cycle and 0.9 mg C/L at the end of the dark cycle, Table S7). DOC characterization by excitation–emission matrix (EEM) fluorescence spectroscopy revealed spectra consistent with fulvic acid-like organics, soluble microbial byproduct-like material, and humic-like compounds in the bioreactors’ water column (Figure S13).^67^

A separate set of batch microcosms amended with the same biomat plus different concentrations of biomat-derived DOC in the water column (Supporting Information Section S3) showed a strong and significant correlation between DOC augmentation and Cu removal (Spearman correlation coefficient = −0.78, *p* < 0.001) but not for Zn removal (Spearman correlation coefficient = −0.24, *p* = 0.20). This negative relationship between the DOC concentration in the water column and Cu removal was observed in both dark and light conditions (Figure S14). Specific ultraviolet absorbance (SUVA_254_)^68^ indicated low aromaticity of biomat-derived DOC with values ranging from 0.012 to 0.032, which are less than those of humic-like compounds derived from soil.^67,69,70^ These findings are consistent with the hypothesis that an increase of bulk DOC concentrations in the water column associated with photosynthetic exudates contributes to diel concentrations of dissolved Cu but not Zn.

### Metal Sequestration and Stability Shift with Biomat Depth

Accumulation of Zn and Cu that were removed from the water column predominantly occurred in the surficial 0–5 mm layer of the biomat within flow-through laboratory bioreactors. Sequential extractions revealed that the largest increases in the concentrations of Zn and Cu were observed in the labile (ACE + LO) phase (Figure 3). Smaller mean differences of the control bioreactor at each biomat depth section compared to those of metal-augmented bioreactors (Table S10) indicated that variations in depth metal profiles resulted from metal addition rather than other experimental factors. Analogous ratios of ACE + LO, oxidizable, and acid-extractable phases were maintained across the first 15 mm of sediment depth (Figure 3a/b right bars). Calcium depth profiles (Figure S15a) were similar to those of Zn and Cu, where the largest changes occurred in the labile fraction of the surficial 0–5 mm layer. This could be due to calcium carbonate (co)­precipitation with Zn occurring at the water–biomat surface, possibly as zinc hydroxide or carbonate precipitates and/or complexes.^71–73^ Past research has revealed that the surficial 5 mm of biomat experiences pronounced diel shifts in DO and transcriptional genes associated with nutrient and xenobiotic attenuation, with less influence at further depths,^21^ supporting observations herein that 0–5 mm is the most reactive layer with respect to water column exchange and constituents. The broader literature on autotrophic and heterotrophic biofilms supports the concept of a millimeter-scale reactive zone at the biomat/water interface for metal removal.^54,74^

In contrast, the deeper 20–25 mm layer was not influenced by experimental aqueous metal augmentation. Consistent with background samples, Cu predominately associated with the oxidizable phase (likely with sulfur species, Table S11), while the majority of Zn was present in the acid-extractable phase. Biomat-associated Al, Fe, Mg, Mn, and Si were strongly correlated with each other (Spearman correlation coefficients >0.7, *p* < 0.05), and the majority of them were in the acid-extractable phases, likely representing clay minerals such as chlorites within the biomat.^27^ These elements were also independent of biomat depth and metal addition (Figure S15). This suggests that surface sorption onto these clay minerals is more likely than coprecipitation/incorporation of Zn/Cu and chlorite, which would alter the distributions of these elements.^75,76^ However, shifts toward a dominance of acid and oxidizable fractions in deeper layers indicate a potential for temporal maturation toward stronger complexes during biomat growth and accretion.^23^ Hence, sequestered constituents in deeper accretion layers are unlikely to be released unless there is a physical perturbation of the biomat sediments.

### Tracking Surficial Metal Sequestration through Stable Isotope Amendments

When focusing on the biomat surficial layer, high background total concentrations of Zn and Cu in the bulk biomat relative to the experimental introduction presented analytical challenges to directly quantify Zn and Cu sequestration. To address this, batch microcosms containing a thin layer (∼10 mm) of the biomat were augmented with Zn and Cu solutions enriched in the heavier isotope (^68^Zn and ^65^Cu). This provides a more sensitive quantification for metal accumulation compared to total metal analysis. This approach resulted in significant increases (*p* = 0.016 by the Kruskal–Wallis test) in isotopic ratios of both Zn and Cu during sequential extractions (Figure 4). As the addition of enriched heavier isotopes were magnitudes higher than natural abundance fractions, previously reported isotopic fractionation effects of Zn and Cu^28,77,78^ were considered negligible in influencing the ratio changes observed in these microcosms.

**Figure 4:**
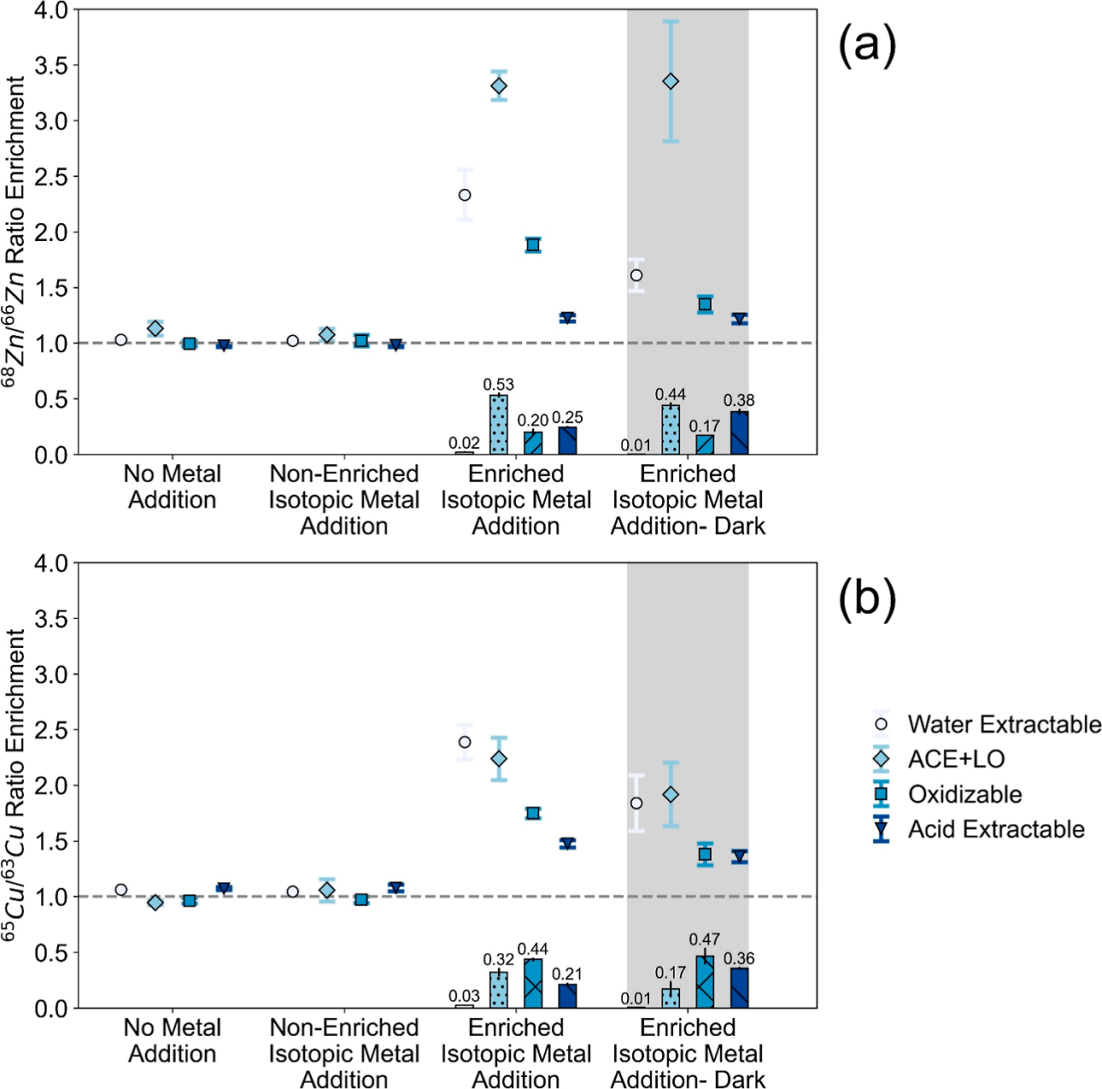
Isotopic ratios of ^68^Zn to ^66^Zn (a) and ^65^Cu to ^63^Cu (b) in each sequential extraction phase in the biomat of each batch microcosm (*n* = 12) at the end of the experiments. The shaded area indicates the microcosms under the dark during the experiments, while all others represent under diel conditions. Enrichment was calculated using eq 1. Gray dotted lines indicate the isotopic ratios at their natural abundance. Histogram bars and the numbers at the bottom indicate the fractions of accumulated zinc/copper in each phase calculated by eqs S5 and S6. The error bars indicate the standard deviation of triplicates.

Within the time frame of these batch experiments (∼116 h), a total of 30.0 ± 8.4 μg/g Zn and 13.3 ± 4.1 μg/g Cu were accumulated in the biomat. This represented 16 ± 3% and 26 ± 5% of the total background of these elements, respectively. The largest increase in isotopic ratio enrichment (eq 1) compared to natural abundance isotopic ratios was present for both Zn and Cu in the labile phases (water-extractable and ACE + LO) of the biomat. In contrast, there was minimal association with the acid-extractable phase (Figure 4), although this could be biased due to the existing high background concentrations of Zn and Cu in the acid-extractable phase. When normalized by accumulated fractions in each phase (eqs S5, and S6), the highest fraction of accumulated Zn still occurred in the ACE + LO phase (53% with light and 44% under dark). In contrast, the highest fraction of accumulated Cu occurred in the oxidizable phase (Figure 4).

A significant correlation was observed in the ACE + LO phase between ^68^Zn and calcium (Spearman correlation coefficients >0.7, *p* = 0.018, Figure S17a), with 21–37% of total calcium accounted for in that phase. This provides further evidence for the association of Zn with calcium carbonate, as discussed in previous sections. For Cu, a correlation was observed in the oxidizable phase between ^65^Cu and Mg, Fe, Ni, and Mn (Spearman correlation coefficients >0.7, *p* < 0.05, Figure S17b). However, less than 15% of total Mg, Fe, Ni, and Mn were accounted for in the oxidizable phase (Table S14). In contrast, much of the total sulfur (58–65%, Table S14) was found in the oxidizable phase. Hence, correlations (Spearman correlation coefficients >0.7, *p* < 0.05, Figure S17b) between sulfur and ^63^Cu and sulfur and ^68^Zn suggest that the background Cu and a smaller fraction (17–20%) of accumulated Zn in the oxidizable phase could be associated with sulfur species.

Consistent with the flow-through bioreactor experiments, we observed higher dissolved Cu concentrations under light (higher pH) and higher dissolved Zn concentrations under dark conditions (lower pH) in the batch microcosms. The relatively constant pH (6.4–7.5) in the dark-only biomat microcosms with 83% and 82% removal of Zn and Cu, respectively, around 24 h (Table S12) further supports the role of sorption in removal. In addition, the no-biomat control microcosms with higher pH (8.9) exhibited 20% removal and a decrease in the isotope ratio enrichment (eq 1) of Zn concentrations in the water column but no change for Cu, suggesting that pH-induced precipitation plays a more pronounced role in Zn dynamics than Cu. Because the water column was >100-fold enriched in the heavier isotopes, the decrease in the isotope ratio enrichment was largely equivalent to the net removal of Zn/Cu from the solution. It could not be used to differentiate the isotope fractionation by active uptake versus by passive sorption.

DOC concentrations in the no-biomat control microcosms were similar under both light (2.6 ± 0.2 mg C/L) and dark (2.1 ± 0.3 mg C/L) incubations. DOC concentrations at the end of the experiments were higher in the biomat microcosms exposed to light (5.3 ± 1.4 mg C/L) than in the dark-only microcosms (2.0 ± 0.3 mg C/L). Analogous to the flow-through bioreactors, these were best characterized as a combination of fulvic acid-like organics, soluble microbial byproduct-like material, and humic-like compounds (Figure S16). In addition, the humification index,^40^ biological index,^41^ and fluorescence index^42^ calculated from respective fluorescence intensities were 0.67–0.74; 0.65–0.80; and 1.72–1.83, with no clear day–night differences (Table S13). These ranges indicate our experimental DOC was more labile than soil-derived humic acid in origin.^41,79^ Hence, batch findings from the enriched isotope microcosms were consistent with the hypothesis that autotrophic DOC exudates increase the stability of Cu in the water column.

### Intertwined Biogeochemical Influences on Copper and Zinc Cycling

Our experimental results from flow-through bioreactors and batch microcosms indicate a series of potentially competing and synergistic mechanisms that influence Zn and Cu diel variations in the water column and sequestration by the photosynthetically active biomat. These mechanisms include precipitation, sorption/surface complexation to different phases (calcium carbonate, iron (oxyhydr)­oxides, clay minerals such as chlorites, organic complexes at the biomat surface, etc.), complexation with DOC in the water column, and microbial uptake/assimilation.^27^ To further query these mechanisms, a geochemical model utilizing PHREEQC was established (details in Supporting Information Section S6; reactions in Table S15). By variation of the input quantity of CO_2_ (aq) to mimic autotrophic consumption and resultant pH increases, output pH values were matched with measured experimental pH values (Figure S18). Influent water chemistry data were integrated as another input to predict the changes in dissolved Zn and Cu concentrations. Mineral phases identified from prior findings^27^ and humic/fulvic substances from WHAM and Model VI in Visual Minteq databases were incorporated into the model (Table S15).^80,81^ Microbial exudate compounds that had existing complexation reaction constants in Visual Minteq databases were added based on studies on *Synechocystis PCC 6803* and other photosynthetic organisms.^82,83^

The modeling effort revealed that biomat microbial organic surface complexation, with strong contributions from microbial phosphoryl groups defined in the previous study (Supporting Information Section S6),^27^ was a dominant contributor to net removal of aqueous Zn and Cu. This is consistent with prior findings at lower metal concentrations that organic components play a more prominent role than inorganic components.^27^ The equilibrium model also supported our observations that different processes influence the diel changes of soluble Zn and Cu. In the model, diel changes of dissolved Zn were influenced by competing mechanisms between microbial organic surface complexation and sorption/coprecipitation with calcite. Although saturation indices for pure zinc hydroxide or zinc carbonates were below zero (Table S17), this does not rule out the possibility of zinc sorption/coprecipitation with calcite.^2,61–63^ The modeled results (Figure 5b) did not fit the trend of our experimental data entirely, suggesting that there might be some limitations in applying existing generic Zn sorption and complexation reaction constants to our system. The model also treats each time point as an equilibrium condition and hence does not capture possible kinetic limitations in zinc coprecipitation with calcite.

**Figure 5:**
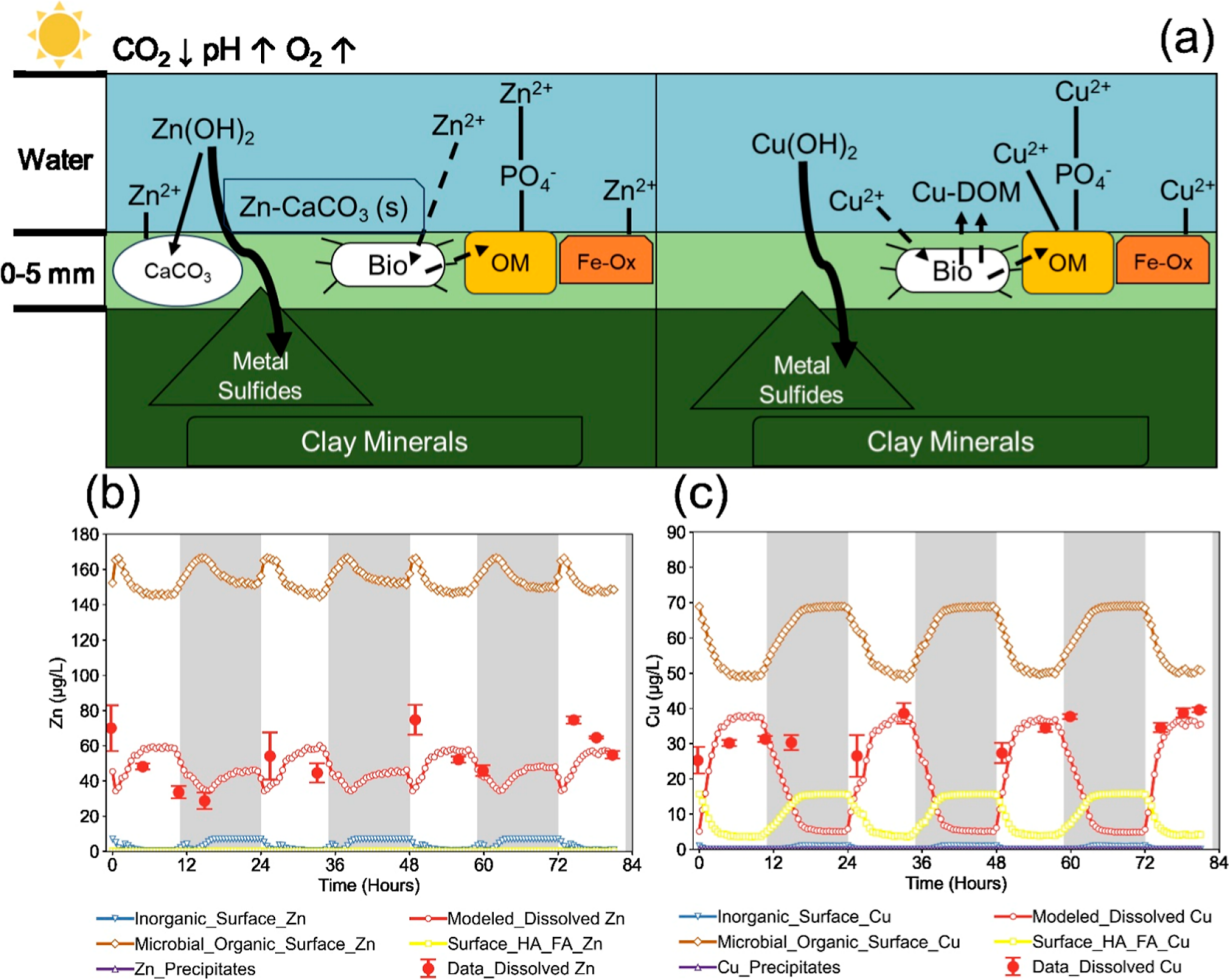
Conceptual model of interactions of Zn and Cu with the biomat in the presence of sunlight (a). Straight lines indicate sorption/surface complexation, dashed lines indicate microbial activities, and curved lines indicate potential transport. The results of the equilibrium modeling are shown in (b) for Zn and (c) for Cu. Red points depict experimental data previously shown in Figure 1d. (D)­OM: (dissolved) organic matter. Fe-Ox: iron (oxyhydr)­oxides. Bio: bacteria, diatoms, and other algae, detailed composition in the text. HA_FA: humic/fulvic substances.

Consistent with the observations in batch enriched isotope microcosms where biomat sorption contributed the most to Cu removal at all pH values, Cu–organic surface complexation with microbial organic groups and humic/fulvic substances strongly influenced the diel behavior of dissolved Cu (Figure 5c), with desorption driving the increase in dissolved Cu under light conditions (photosynthesis). Since quantitative information about the exact molecular nature of Cu-DOC complexes or Cu-organic surface complexes in our system was limited, the reaction constants from databases used for generic Cu-humic/fulvic substance surface complexation might not be the most accurate in the model, which could result in over- or underestimation. However, the modeled increase aligns well with the observed increase in dissolved Cu in the experiments.

Metal assimilation could also play a role in Cu and Zn attenuation. Both are trace micronutrients for photosynthesis and serve as cofactors for metabolic activities previously identified in UPOW wetlands such as denitrification^21^ and methane oxidation.^26^ Analysis of field-derived UPOW biomat metatranscriptome data^21,26^ (Figure S12) revealed the presence of transcripts encoding genes for copper-specific transport/uptake/resistance—*copABC*, *copR*/*cusR*, *copZ*, *cueR*, etc. (complete search criteria described in Supporting Information S2.2),^56^ zinc-specific transport/uptake/resistance (*zntABC*, *znuC*, etc.),^84,85^ and divalent metal uptake/resistance (*cusAB*, *czcABC*)^84,86^ in the surficial 0–5 mm biomat at both sunset and just before sunrise. While zinc and copper exposure in the field-scale system (<20 μg/L for Zn and <3 μg/L for Cu) were below ecotoxicological benchmarks by USEPA,^87^ these genes are widely reported in a diversity of sources such as sediments, wastewater, and mine drainage.^84,86,88–90^ They are also part of the regulation system of *Synechocystis PCC 6803* and other photosynthetic organisms.^56,91,92^ These processes were not captured by PHREEQC modeling, and the magnitude of this impact was unknown. Because of the difficulty in distinguishing between zinc-specific and copper-specific genes, in addition to ancillary factors, the possible diel differences in microbial assimilation as a result of the expression of these genes are difficult to resolve. Rather, microbial-derived functional groups and DOC complexation with microbial exudates incorporated into the model could be considered as a macroscopic approximation for some of these processes.

Inverse Zn and Cu trends are counter to absolute solubility interpretations as a function of carbonate and hydroxide precipitation as these would decrease the solubility of both elements when pH increases. However, this paradox has been documented in certain natural rivers and creeks,^5,7^ with a parallel observation of shallow waters containing benthic photosynthetic sediments. To better understand this within a broader environmental context, we compiled and analyzed diel changes of dissolved Zn and Cu in studies that also reported similar diel changes of DO and circumneutral to basic pH with proposed mechanisms (Table 1).^2–5,7,8,10,13,28,54,59,60,66,93,94^ This analysis revealed that reported diel trends of dissolved Zn were universally consistent and could be driven by a combination of precipitation and sorption processes when the dissolved Zn concentrations were above 2 μg/L. Lower concentrations of dissolved Cu (<10 μg/L)^2,5,7,8,10,12,13,66,93^ were associated with either no clear diel changes or an inverse trend to that of Zn. In contrast, when concentrations of dissolved Cu were in excess of 50 μg/L^2,3^ (above most ecotoxicological benchmarks^87^), diel shifts in solubility were analogous to that of Zn. In our laboratory bioreactors, although the introduced concentrations of Cu (inlet ∼100 μg/L) were in excess of some literature examples in Table 1, the strong net removal by organic moieties within the biomat decreased observable water column Cu concentrations to 25–40 μg/L, lower than the range observed in other natural environments where Cu showed analogous diel solubility changes to Zn. However, this concentration-related shift is further dependent on site-specific net removal processes, such as competing cations and surface areas for sorption. Measurements that capture or characterize these variables could be used to further refine predictions of copper behavior and attenuation capabilities across a more diverse set of natural environments.

**Table 1:** Compilation of Studies on Diel Changes of Dissolved Oxygen and Circumneutral to Basic pH, Dissolved Cu and Zn, and Dissolved Organic Carbon^a^

	Diel changes of parameters (generally from sunrise to sunset, unless specified otherwise)							
locations	pH	dissolved oxygen (mg/L)	*T* (°C)	dissolved Cu (μg/L)	dissolved Zn (μg/L)	dissolved organic carbon (mg/L)	proposed mechanisms for diel changes of dissolved Cu and Zn	references
High Ore Creek, MT	8.12 to 8.34	NR	11.5–21	3 to 4.3*	634 to 214	NR	Cu: unresolved; Zn: sorption, precipitation with calcite	^2^
Lower Daisy Creek, MT	7.49 to 7.83	NR	6.9–17.3	55.4 to 34.3	112 to 45	NR	Cu: unresolved; Zn: sorption, precipitation with calcite	^2^
Fisher Creek F3, MT	6.8*	NR	10–17	100 to 50	45 to 35	NR	Cu and Zn: sorption to Fe-Ox	^3^
Prickly Pear Creek, MT	8 to 8.5	NR	14–22	NR	52.4 to 13.1	NR	Cu: NR; Zn: sorption to Fe-Ox	^4^
Riou Mort, France	7.7 to 8.5	73% to 130% saturation (sunset to mid-day)	13–18	2.43 to 3.46	NR	1.4 to 4.5	Cu: complexation with organic matter; Zn: NR	^5^
Eagle River near Minturn, CO	7.8 to 8.2	8.0 to 8.8	8.9–12.6	0.99 to 1.4	63 to 38.3	1.54 to 1.35*	Cu: unresolved; Zn: sorption to Mn phases	^7^
Eagle River below Milk Creek near Wolcott, CO	8.0 to 9.3	6.2 to 11.8	11.9–19.3	0.98 to 1.4	5.5 to <4	1.65 to 1.84	Cu and Zn: contribution of algal growth and sorption to Mn phases	^7^
Clark Fork River, MT	8.2 to 8.7	76% to 120% saturation (sunrise to mid-day)	15–26	6.4*	19.5 to 6.5	NR	Cu: no diel change; Zn: sorption to Mn phases	^8^
Catalina Island, CA	NR	NR	18.8–19.4	0.15 to 0.08*	NR	NR	Cu: sorption to organic matter	^10^
Prado wetlands, CA	7 to 9	2 to 20	14–30	1 to 2.5	15 to 7.4	3.75 to 4.03	Cu and Zn: sorption	^12^
Lab flow-through system with a phototrophic biofilm	6.9 to 9.8	5 to 20	18–23	44.8 to 30.1	2015 to 45.5	0.75 to 1.1	Cu and Zn: complexation and sorption with organic matter, active uptake	^28^
Lab flow-through column with a biofilm	6.5 to 10	0.16 to 32	15	32*	NR	NR	Cu: no diel change; Zn: NR	^54^
Silver Bow Creek at Miles Crossing, MT	7.5 to 9.6	NR	12–22	13 to 9.9	89 to 8.2	4.6*	Cu and Zn: unresolved	^59^
Clark Fork River, MT	8.2 to 8.7	4.8 to 12.8	15–26	BDL	8 to 2	NR	Cu: BDL; Zn: sorption to redox formed Fe-Ox and Mn phases	^60^
Lake Svyatoe, Northern Eurasia	7.4 to 8.4	8 to 12	28–30	0.5*	1.5 to 0.25*	13.5*	Cu and Zn: unresolved	^66^
High Ore Creek, MT	8.28 to 8.45	7.5–10.25 mg/L (sunset to sunrise)	7–20	1.94 to 3.35*	700 to 350	3.1 to 3.3*	Cu: no diel change; Zn: unresolved	^93^
Lambert Run constructed wetland, WV	7.09 to 7.26	NR	19.7–25.4	NR	50 to 35	NR	Cu: NR; Zn: sorption to Fe-Ox	^94^
Fresh wetland ponds, UT	8.3 to 9.3	5 to 16	20–30	3*	NR	13*	Cu and Zn: no diel change	^13^

a NR: not reported. BDL: below the detection limit. *: No or no clear diel change. Fe-Ox: iron (oxyhydr)­oxides.

Much of the aforementioned literature did not report DOC values. However, a select subset did report a daytime increase and a nighttime decrease of DOC and suggested a potential role in this association with copper.^5,7,28^ However, Cu diel patterns in nature could be attributed to other variables beyond just DOC, pH, or sorptive mineral phases,^2,17^ so the above relationship between Cu concentrations and diel patterns may be most suitable in analogous biomass-rich photosynthetic sediments such as wetlands and periphyton found in rivers and streams.

## Environmental Implications

Laboratory flow-through bioreactors containing field-derived materials provide opportunities for hypothesis-driven research to explore biogeochemical reactions of interest in UPOW constructed wetlands and analogous natural systems containing periphyton and photosynthetic sediments. In these systems, photosynthesis increases the pH and, in turn, enhances zinc sorption and possible coprecipitation with calcite across environmentally relevant concentrations. In contrast, copper biogeochemistry is more complex, with prominent influences from organic associations with photosynthetic sediments and DOC ligand effects that increase solubility. The resultant understanding of dynamic mechanisms associated with photosynthetic diel cycling of zinc and copper is hence important for monitoring, designing, and managing decisions that better protect nature-based and ecological resources. A possible outcome is the more active management of constructed wetlands, which are traditionally passive treatment systems. Automated equipment could be applied toward sampling campaigns that capture diel dynamics to understand and predict temporal shifts in water quality concerns. Changes in the bioavailability of dissolved Cu during the day versus night depend on the nature of Cu complexes as Cu-humic complexes could be less bioavailable, whereas Cu complexes with lower molecule weight organic matter could be more bioavailable to aquatic organisms.^95^

Integration of these processes could be applied to more holistically understand and mitigate copper ecotoxicity and potentially construct and manage wetlands that optimize metal­(loid) attenuation. For example, wetland sediments could be augmented toward an optimal ratio of inorganic and organic materials. Hydraulic manipulation informed by diel cycles could be applied to maximize the metal attenuation windows. Precipitation and microbial assimilation along with sedimentation could contribute to the long-term accumulation of Zn and Cu in the sediments. Strategic harvesting of the biomat, which accretes at approximately 1–2 cm per year in the field,^25,33^ could be applied to enhance long-term metal attenuation, limit hazardous waste accumulation, and potentially support resource recovery in this more actively managed scenario.

Field and laboratory data were integrated with equilibrium modeling to develop a conceptual framework that can be applied to predict Zn and Cu diel changes and removal. This framework was then used to validate existing experimental data and provide new insights into the relative contributions of abiotic and biotic reactions, including sorption/coprecipitation with calcium carbonate and iron minerals, complexation with DOC in the water column and at the surface, precipitation, and microbial uptake/assimilation. This approach can inform a toolkit and framework for understanding removal mechanisms in analogous systems and help shortlist the more prominent processes that influence metal diel changes. As thermodynamic modeling relies on existing reaction constants and databases, more characterization of biological complexes and surfaces is needed for a more complete representation of possible diel cycling mechanisms. Because flow-through bioreactor experiments were conducted under controlled laboratory conditions, other environmental factors that could affect diel cycling in the field, such as hydrological variables, should be considered. Given that diel cycling is a widely observed phenomenon in photosynthetic environments, future work can build on this foundation using variables such as pH, DO, temperature, metal concentrations, DOC, other essential elements (Fe, Mn, Ca, Mg, etc.), and broad hydrological parameters (flow rate, hydraulic residence time) to develop a more complete biogeochemical model that incorporates the complex physical, chemical, and biological interactions governing zinc and copper behavior in constructed wetlands.

## Supplementary Material





## Notes

Flow-through bioreactor setups and sampling procedures; calculations of removal rate models; metadata for field microbial samples, amplicon sequencing procedures and bioinformatics; analytical methods for all analytes; characterization of dissolved organic matter; supporting data for depth changes of metal phases; batch isotope microcosm setups; analytical methods for isotope detection and correction; calculations for isotopic ratio enrichment and accumulation; and PHREEQC model setups for diel changes (PDF)
Detailed data for flow-through bioreactor experiments and batch isotope microcosms; sequential extraction procedures; 16*S*/18S rRNA sequencing sample list; and major reactions used in PHREEQC models (XLSX)

## #

The Ecosystems Center, Marine Biological Laboratory, Woods Hole, Massachusetts, 02543, United States

## ¶

School of Civil and Environmental Engineering, Cornell University, Ithaca, New York, 14850, United States.

## ∇

Department of Geography and Environmental Engineering, United States Military Academy, West Point, New York, 10996, United States.
