# Supplementary material for: Photosynthetic Diel Cycling Influences Inverse Zinc and Copper Solubility Dynamics within a Constructed Wetland
Source: Environ Sci Technol. 2026 Apr 2;60(14):10775–88. doi: 10.1021/acs.est.5c12039 (PMC13085814; doi:10.1021/acs.est.5c12039)
Supplement: Supplementary file 1 [file es5c12039_si_001.pdf]

Supporting Information for:

Photosynthetic diel cycling influences inverse zinc and copper solubility dynamics within a constructed wetland

Zhaoxun Yang[1,2,3], Gary F. Vanzin[1,2], Michael A. P. Vega[1,4], Adam R. Brady[1,5], Lino Morales Paredes[2,6], James F. Ranville[2,7] and Jonathan O. Sharp[1,2,8,]*

AUTHOR ADDRESS

[1] Department of Civil and Environmental Engineering, Colorado School of Mines, Golden, Colorado, 80401, United States

[2] Center for Mining Sustainability (https://miningsustainability.mines.edu), Colorado School of Mines, Golden, Colorado, 80401, United States

[3] Present address: The Ecosystems Center, Marine Biological Laboratory, Woods Hole, Massachusetts, 02543, United States

[4] Present address: School of Civil and Environmental Engineering, Cornell University, Ithaca, New York, 14850, United States

[5] Present address: Department of Geography and Environmental Engineering, United States Military Academy, West Point, New York, 10996, United States

[6] Departamento Académico de Química, Facultad de Ciencias Naturales y Formales, Universidad Nacional de San Agustín de Arequipa, Arequipa, 04001, Peru

[7] Department of Chemistry, Colorado School of Mines, Golden, Colorado, 80401, United States

[8] Hydrologic Science and Engineering Program, Colorado School of Mines, Golden, Colorado, 80401, United States

Number of Pages: 33

SI Figures: 19

SI Tables: 0

Table of Contents

S1. Flow-through bioreactor experiments setup and results

S2. Supplementary information for field-bioreactor associations

S2.1 Calculations of removal rate models

S2.2 Metadata for field microbial samples, amplicon sequencing procedures, and bioinformatics

S2.3 Supplementary results

S3. Methods for other aqueous analytes, characterization of organic matter, and results

S4. Supplementary information for depth changes of metal phases

S5. Supplementary information for batch isotope microcosms

S5.1 Experimental set-ups

S5.2 Analytical methods for isotope detection and correction

S5.3 Calculations for isotopic ratio enrichment and accumulation

S5.4 Supplementary results for batch isotope microcosms

S6. PHREEQC models for diel changes

Section S1. Flow-through bioreactor experiments setup and results

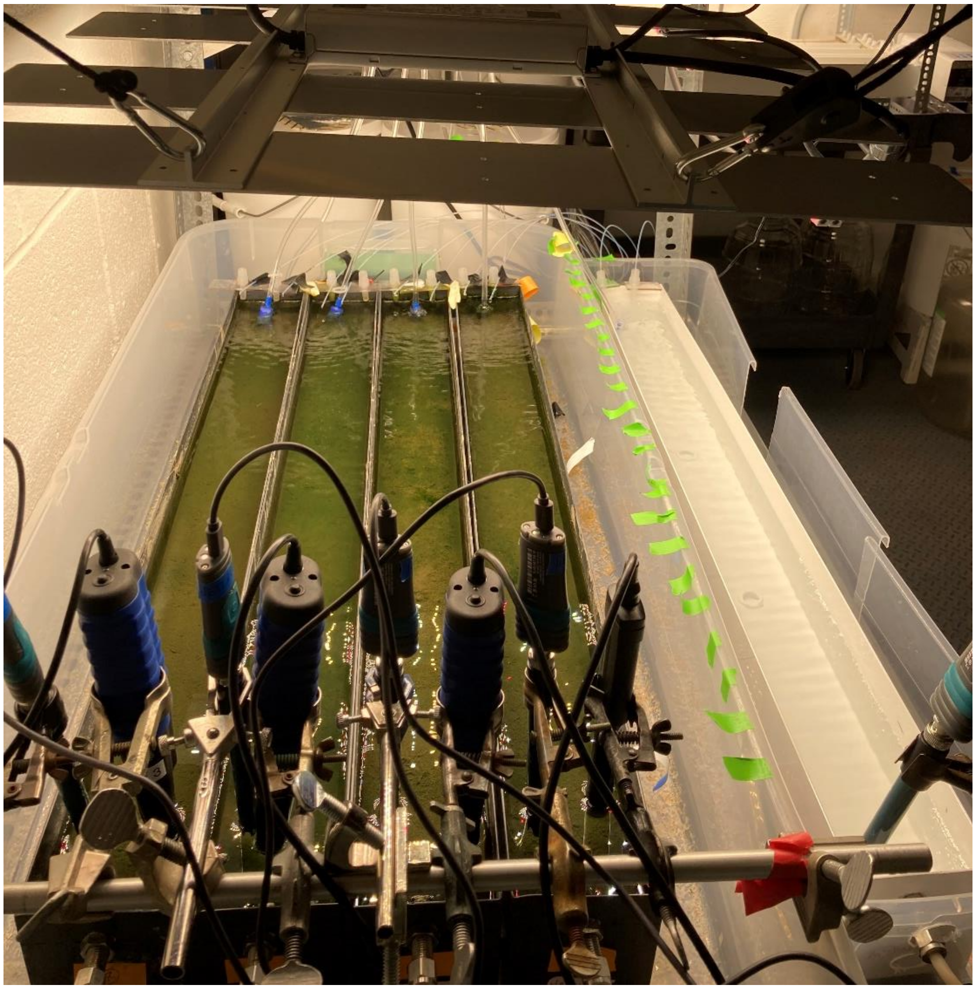

Figure S1. Flow-through bioreactor experimental setup. From left to right: Bioreactor 4, Bioreactor 3, Bioreactor 2, Bioreactor 1, No biomat control Bioreactor 5.

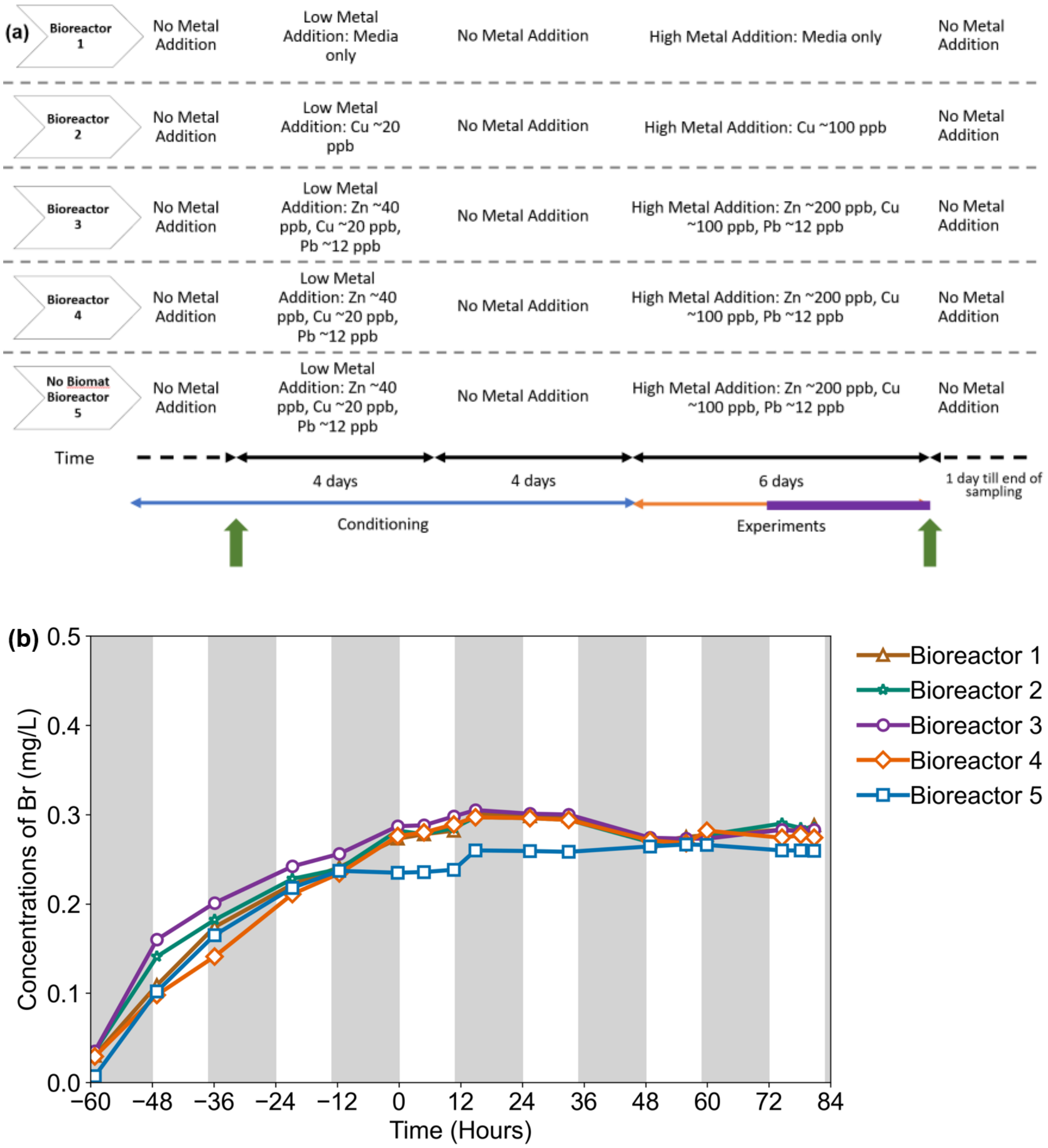

Figure S2. Experimental matrix of flow-through bioreactor experiments. (a) The thick green arrows indicate the two sampling time points of the biomat cores taken for core section geochemical extractions and 16S/18S rRNA sequencing. The thick purple line indicates the time window for data plotted in Figure 1 and Figure S4 when the tracer Br concentrations (b) showed influent equilibrated with the water column in the bioreactors (defined as time zero), *i.e.,* Br concentration in the bioreactor water ~= Br concentration in the influent media (data for the full-time window shown in Table S4). No Metal Addition: only diatom media was added as the influent media. All metal additions were in complement to the diatom media (recipe see Table S2).

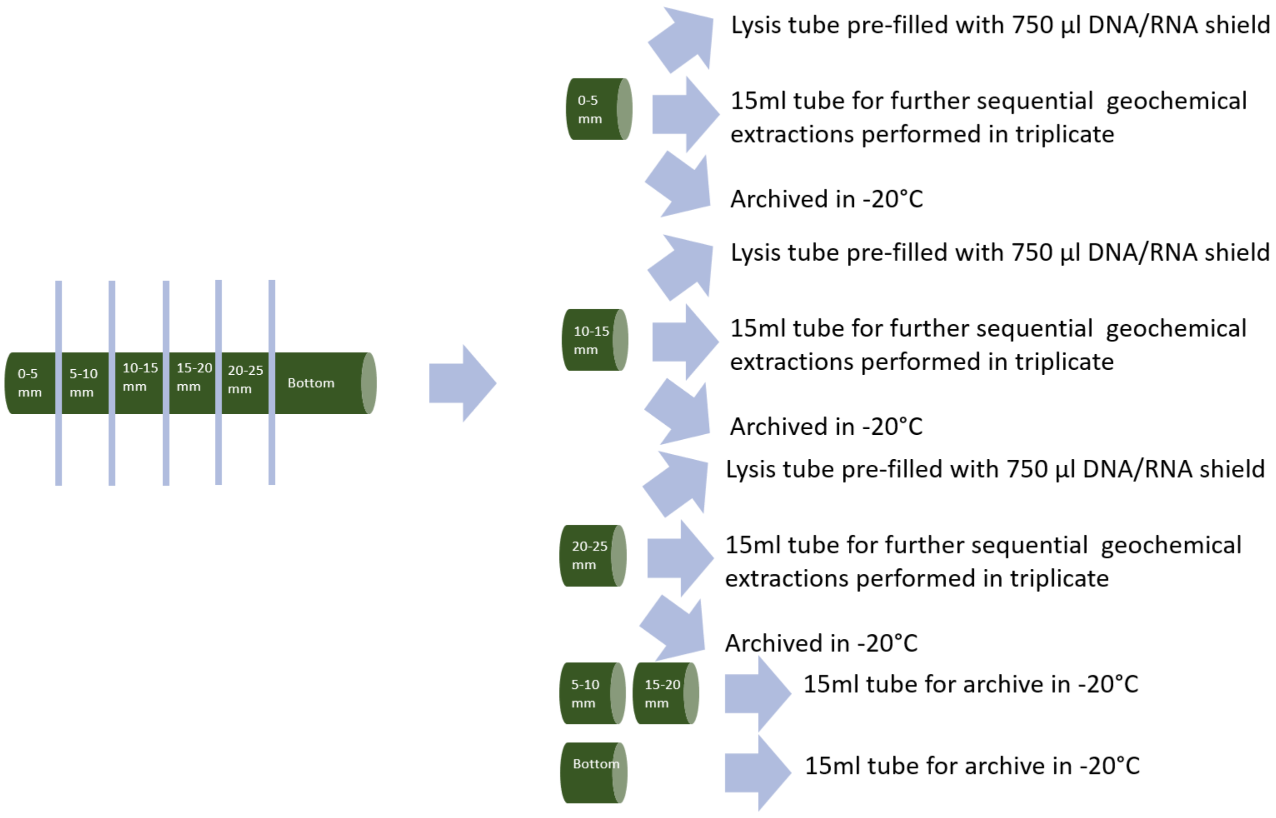

Figure S3. Core sectioning and corresponding aliquots described in the flow-through bioreactor method section.

Biomat cores were sampled from the bioreactors at the beginning and the end of experiments, flash froze in liquid nitrogen, and subsequently processed as sterile 5mm sections using a razor blade and partitioned into three units for 16S/18S rRNA sequencing, geochemical sequential extractions, and an additional archive shown in Figure S3 and previous studies.[1] A frozen core was placed on a flat container surface on ice with a ruler in a laminar flow hood, a razor blade was used to cut each 5mm section, and a tweezer was used to transfer the biomat section from the surface to the corresponding aliquot tubes. 70% ethanol was used to clean the sectioning area and tools before starting each new core. Sections of 0-5mm, 10-15mm, and 20-25mm were divided into three aliquots, one transferred to a lysis tube pre-filled with 750 µl DNA/RNA shield (Zymo Research, Inc.) for 16S/18S rRNA gene sequencing and kept at -80°C

until use, one transferred to a 15ml tube for further sequential geochemical extractions performed in triplicate, and the rest archived. Sections of 5-10mm and 15-20mm were combined into a 15ml tube and the rest of the depth sections were combined into another 15ml tube.

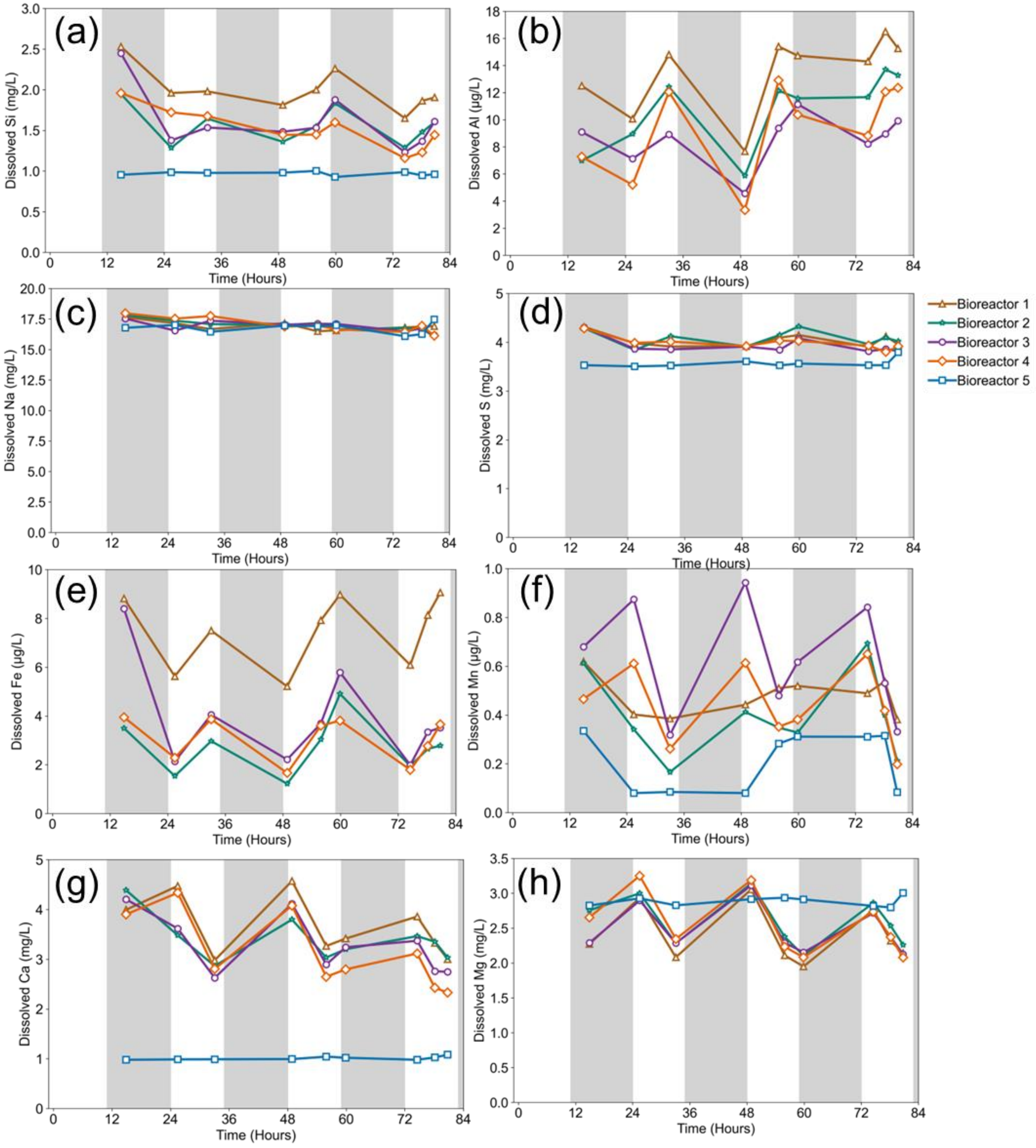

Figure S4. Diel changes of the dissolved concentrations of selected elements in the bioreactors. The plots did not show the Al (b) nor Fe (e) data for Bioreactor 5 because they were below the detection limits after ~5-fold dilution. The corresponding data of are shown in Table S6.

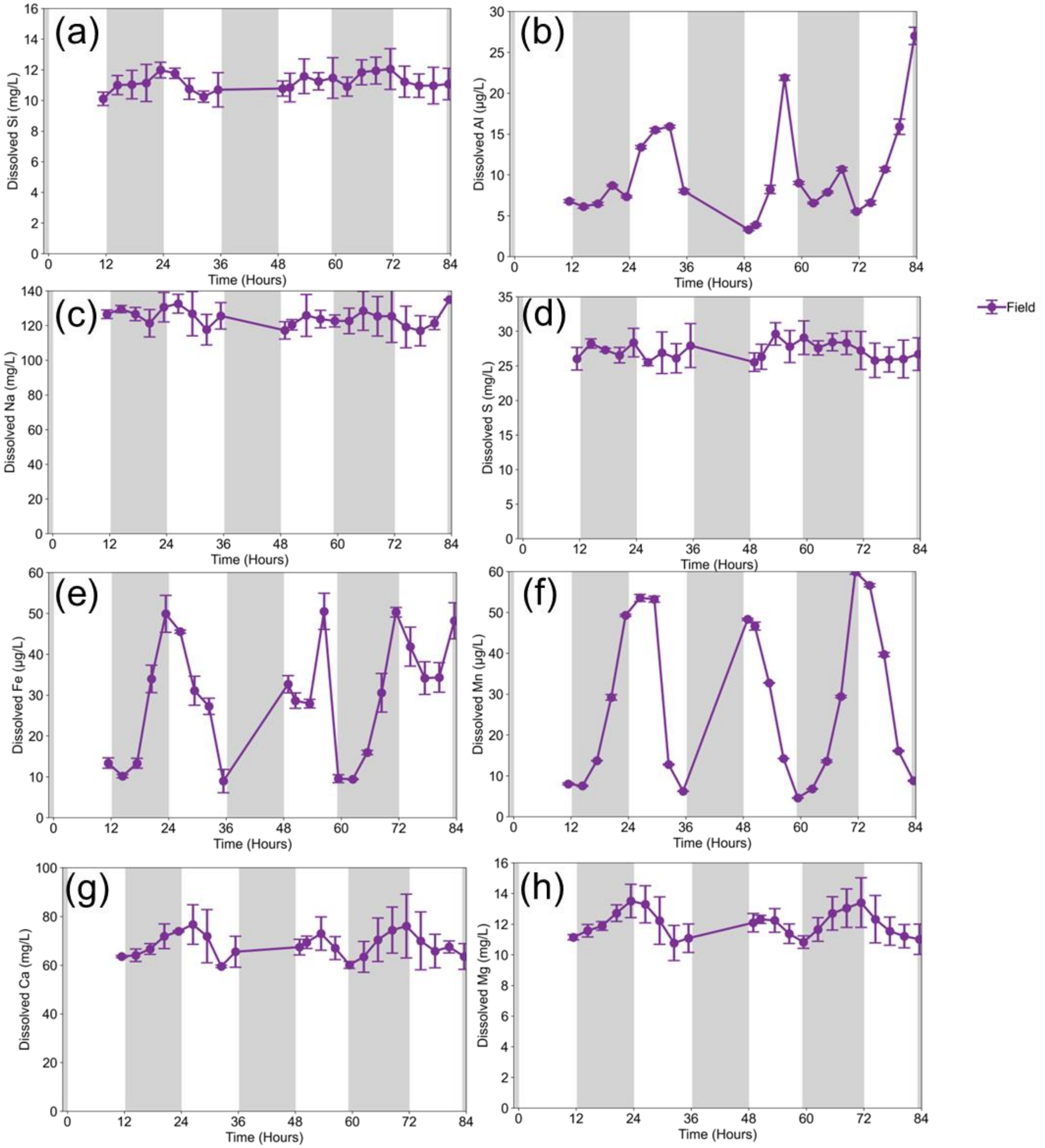

Figure S5. Diel changes of the dissolved concentrations of selected elements in a field-scale UPOW wetlands (data from Reed 2019).

S2. Supplementary information for field-bioreactor associations

S2.1 Calculations of removal rate constants

The tanks-in-series model was used to calculate the rate constant of removal during light and dark time in both the field Prado wetlands and lab-scale bioreactors:[2, 3]

$$\frac{C_{out}}{C_{in}} = (1 + \frac{kA}{NQ})^{-N} \qquad (Eq. S1)$$

where Cout is the dissolved zinc/copper concentration in the bioreactor water near the outlet, Cin is the inlet zinc/copper concentration, k is the areal removal rate constant (m per year), A is the area of the wetland or the bioreactor (m^2), Q is the influent flow rate (m^3 per year), and N is the number of tanks-in-series used to describe the wetland hydraulics. Parameters used for calculations are shown in Table S8.

S2.2 Metadata for field microbial samples, amplicon sequencing procedures, and bioinformatics

The biomat samples were transferred to lysis tubes and bead-beat for 2.5 min (Biospecs Minibeadbeater). DNA extraction was performed using Zymo Research DNA Mini-prep kits, and the eluted DNA was stored in 1.7 ml tubes at -20°C until use. Demultiplexed 16S/18S rRNA sequencing forward and reverse reads of field cores from 2018 (n=40) were downloaded from NCBI BioProject PRJNA818364. All samples used in the analysis were listed in Table S9 (for Figure 2 and Figures S6-S12). None of the negative controls - core sectioning controls (n=2), extraction controls (n=2), and PCR controls (n=2) had quantifiable DNA via Qubit after extraction.

Bioinformatic pre-processing procedures were described in detail in the previous study.[1] In short, samples were demultiplexed by AdapterRemoval2 (v2.3.2)[4] and imported into R (v4.1.2) for DADA2 (v1.22.0) pipeline.[5] Sample raw reads were filtered and trimmed, denoised, merged, and chimeras were removed.[5] Two modifications were made to reach the optimal merging: in addition to manually trimming the primers, 10 forward reads and 4 reverse reads were also trimmed; and in the merging step, minOverlap was changed from 12 (default) to 5. Prokaryote taxonomy assignments were performed on High Performance Computing platforms of Colorado School of Mines based on SILVA database v138.1, and eukaryotes on SILVA database v132.[6] Phylogenic tree alignment was conducted in QIIME2 (v2021.11).[7] The R packages phyloseq (v1.38.0),[8] ampvis2 (v2.7.17),[9] DESeq2 (v1.34.0),[10] ComplexHeatmap (v2.15.2) [11], microbiome (v1.16.0)[12] were used in data processing and visualization.

In order to compare the similarities in the microbial community composition between field and lab bioreactor samples from 16S/18S rRNA sequencing results (Figure 2), we used the vegan::adonis2 test[13] (permutational multivariate analysis of variance method) calculated by Bray-Curtis distance for rarefaction (16S: R^2=0.249, p=0.001, 18S: R^2=0.468, p=0.001); total sum scaling (16S: R^2=0.267, p=0.001, 18S: R^2=0.476, p=0.001); cumulative sum scaling (16S: R^2=0.247, p=0.001, 18S: R^2=0.422, p=0.001) and weighted-UniFrac distance for rarefaction (16S: R^2=0.399, p=0.001, 18S: R^2=0.549, p=0.001); total sum scaling (16S: R^2=0.411, p=0.001, 18S: R^2=0.552, p=0.001); cumulative sum scaling (16S: R^2=0.288, p=0.001, 18S: R^2=0.598, p=0.001). Permutation test on the beta dispersions calculated from Bray-Curtis distance showed no dispersion among groups (p=0.787). Similar ordination patterns were observed regardless of the normalization or rarefaction methods used (Figure S6). Differential abundance analyses between field and lab bioreactor samples were performed by DESeq2 (v1.34.0).[10] Before

creating DESeq objects, amplicon sequence variants (ASV) were clustered at the genus or deepest classifiable level, and filtered to retain only ASV clusters containing at least 10 reads in at least two samples. The results of differential abundance analyses were shown as Log2FoldChange (Figure S7, S11).

In addition, the above methods were used to compare different depth lab bioreactor samples: Bray-Curtis distance for rarefaction (16S: R^2=0.020, p=0.339, 18S: R^2=0.011, p=0.922); total sum scaling (16S: R^2=0.020, p=0.368, 18S: R^2=0.011, p=0.924); cumulative sum scaling (16S: R^2=0.020, p=0.355, 18S: R^2=0.02, p=0.687) and weighted-UniFrac distance for rarefaction (16S: R^2=0.035, p=0.074, 18S: R^2=0.01, p=0.812); total sum scaling (16S: R^2=0.035, p=0.077, 18S: R^2=0.01, p=0.843); cumulative sum scaling (16S: R^2=0.041, p=0.02, 18S: R^2=0.013, p=0.726). No significant differences for 16S rRNA nor 18S rRNA were observed when evaluated at Log2FoldChanges across bioreactor samples of different depths.

The above methods were also used to compare lab bioreactor samples with or without metal addition: Bray-Curtis distance for rarefaction (16S: R^2=0.089, p=0.005, 18S: R^2=0.048, p=0.066); total sum scaling (16S: R^2=0.097, p=0.005, 18S: R^2=0.05, p=0.064); cumulative sum scaling (16S: R^2=0.109, p=0.001, 18S: R^2=0.074, p=0.002) and weighted-UniFrac distance for rarefaction (16S: R^2=0.113, p=0.01, 18S: R^2=0.066, p=0.068); total sum scaling (16S: R^2=0.113, p=0.012, 18S: R^2=0.064, p=0.068); cumulative sum scaling (16S: R^2=0.138, p=0.005, 18S: R^2=0.071, p=0.024). Only one significant decrease ($p<0.001$) with |Log2FoldChange| =7.2 was observed in Genus *Sphingopyxis* in the bioreactor samples after metal addition.

In addition, we utilized a previously constructed metatranscriptome database that consisted of triplicate biomat core samples collected in the field at sunrise and sunset, sectioned into three 5 mm increments of 0-5, 10-15, and 20-25 mm.[1, 14] Sequencing procedures and

bioinformatics were described in detail in Vega et al, 2022, 2023. In the database, we searched for transcripts belonging to zinc and copper-specific genes in the surficial 0-5mm metatranscriptomes at sunrise and sunset (n=6, Figure S12, Table S16). For zinc-specific genes, we searched the "kegg_id" and "kegg_hit" outputs by stringr::str_detect with the following strings: "K01534" "K13638" "K16074" "K09815" "zinc" "Zinc" "Zn" "zn". For copper-specific genes, we searched with: "K07665" "K17686" "K07223" "K19591" "K07810" "K07213" "K01533" "K22552" "K07245" "Copper" "copper" "Cu", and manually removed unrelated genes found due to the broad "Cu" string search based on gene descriptions. For genes encoding for divalent metal resistance (*cusAB*, *czcABC*), we searched with "K15726" "K07787" "K15727" "K07798" "K15726" "czcA" "CzcA" "czcB" "CzcB" "czcC" "CzcC".

S2.3 Supplementary results

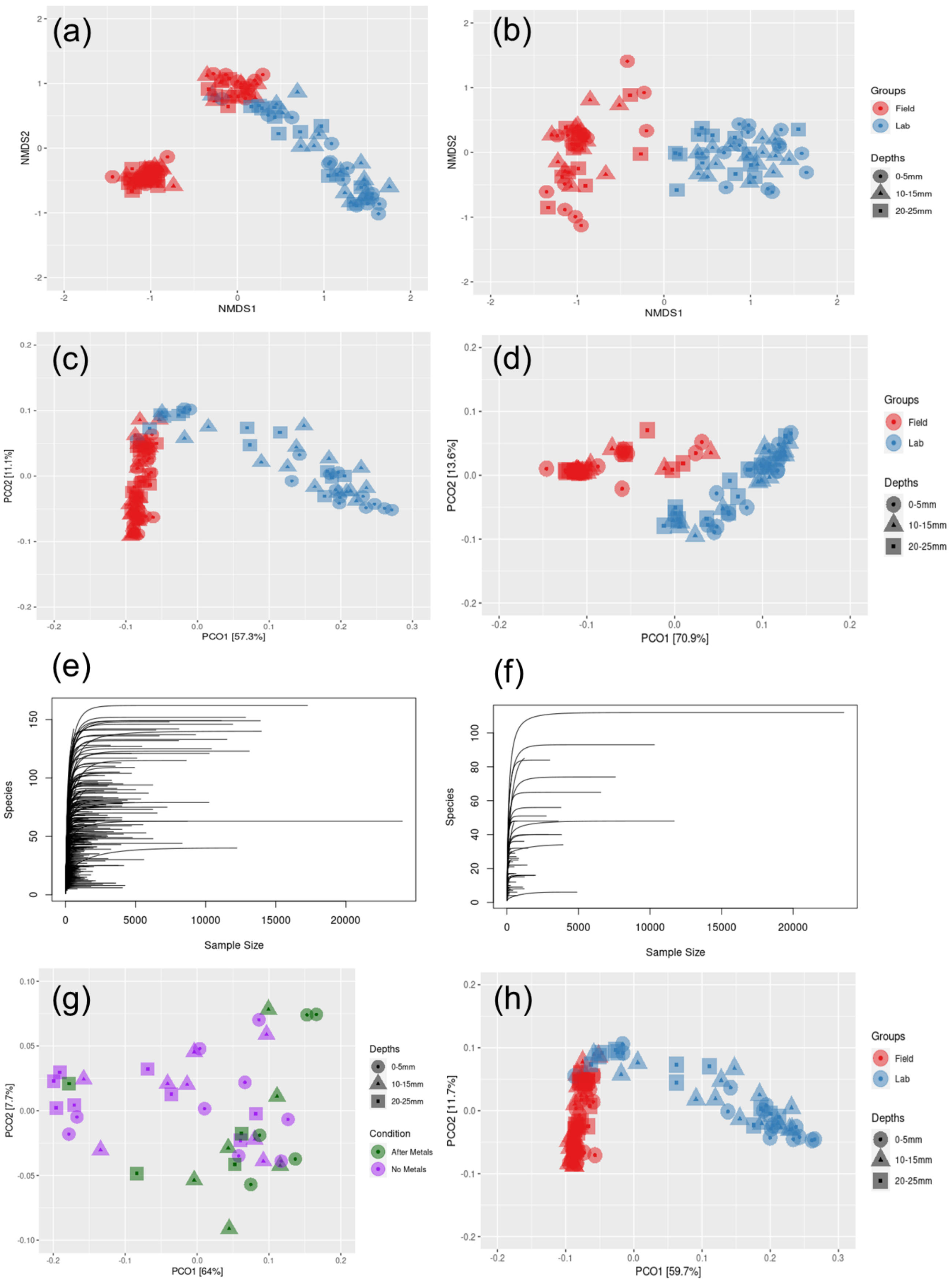
(a)
NMDS1
NMDS2
(b)
NMDS1
NMDS2
Groups
Field
Lab
Depths
0-5mm
10-15mm
20-25mm
(c)
PCO1 [57.3%]
PCO2 [11.1%]
(d)
PCO1 [70.9%]
PCO2 [13.6%]
Groups
Field
Lab
Depths
0-5mm
10-15mm
20-25mm
(e)
Species
Sample Size
(f)
Species
Sample Size
(g)
PCO1 [64%]
PCO2 [7.7%]
Depths
0-5mm
10-15mm
20-25mm
Condition
After Metals
No Metals
(h)
PCO1 [59.7%]
PCO2 [11.7%]
Groups
Field
Lab
Depths
0-5mm
10-15mm
20-25mm

Figure S6. Ordination plots of comparisons between field and lab bioreactor biomat microbial community composition from 16S and 18S rRNA sequencing by non-metric multidimensional scaling (NMDS) and principal coordinate analysis (PCoA). Rarefaction normalization (to 1503 reads for 16S, to 300 reads for 18S) was used. a: NMDS, 16S; b: NMDS, 18S; c: PCoA, 16S; d: PCoA, 18S. Rarefaction curve for all samples: e: 16S; f: 18S. g: PCoA, 16S, grouped by before and after metal addition. No rarefaction was used for h: PCoA, 16S.

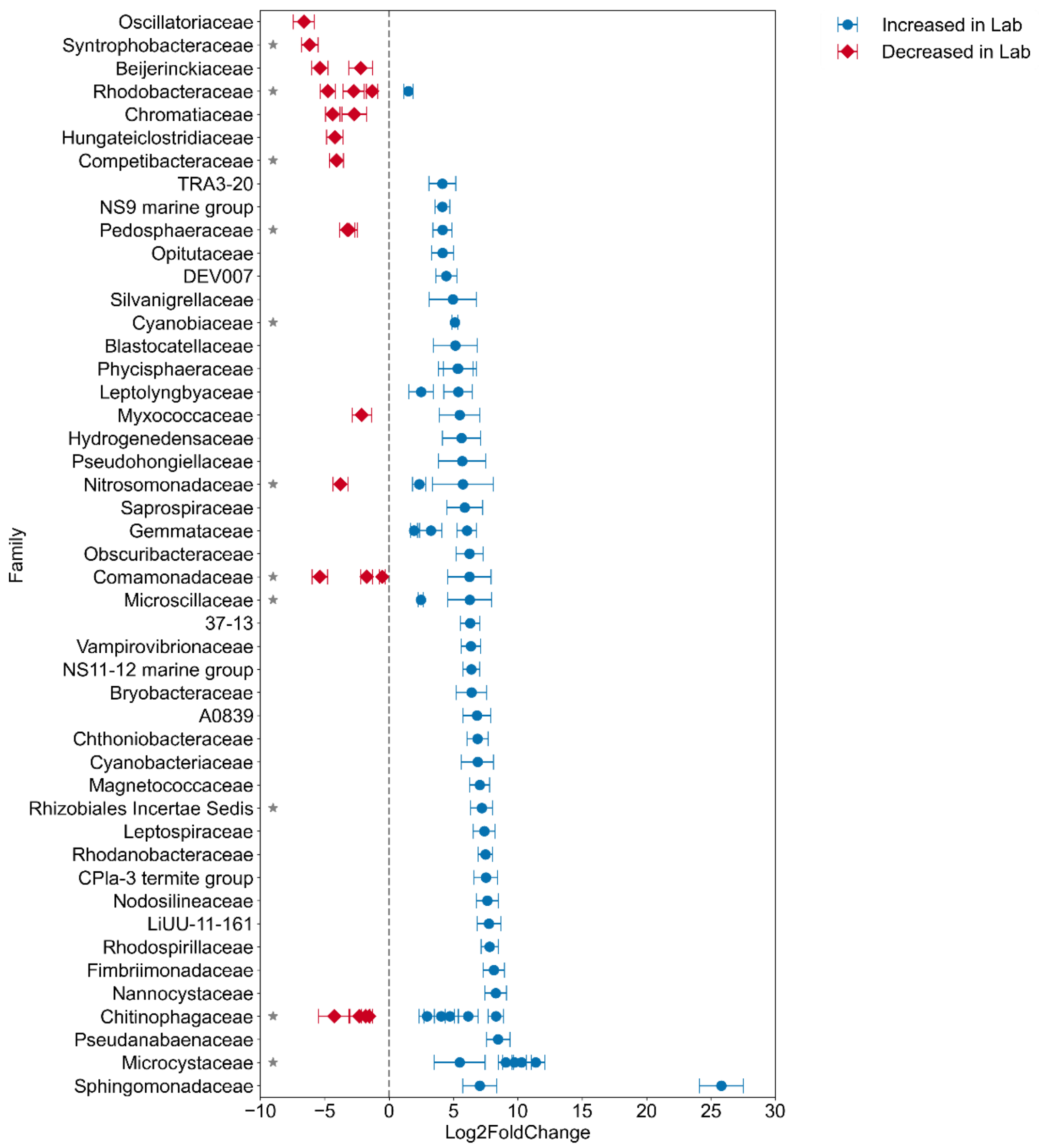

Figure S7. Differential abundance analysis between field and lab bioreactor biomat 16S rRNA samples. Only |Log2FoldChange| > 4 and significant differences ($p<0.05$) were shown. The y-axis denotes the family level taxa with grey stars indicating that the taxa were found in the top 30 taxa of the microbial community, blue circles indicate that the taxa were more enriched in lab bioreactor samples than field samples and red diamonds indicate that the taxa were more enriched in the field than in lab bioreactor samples.

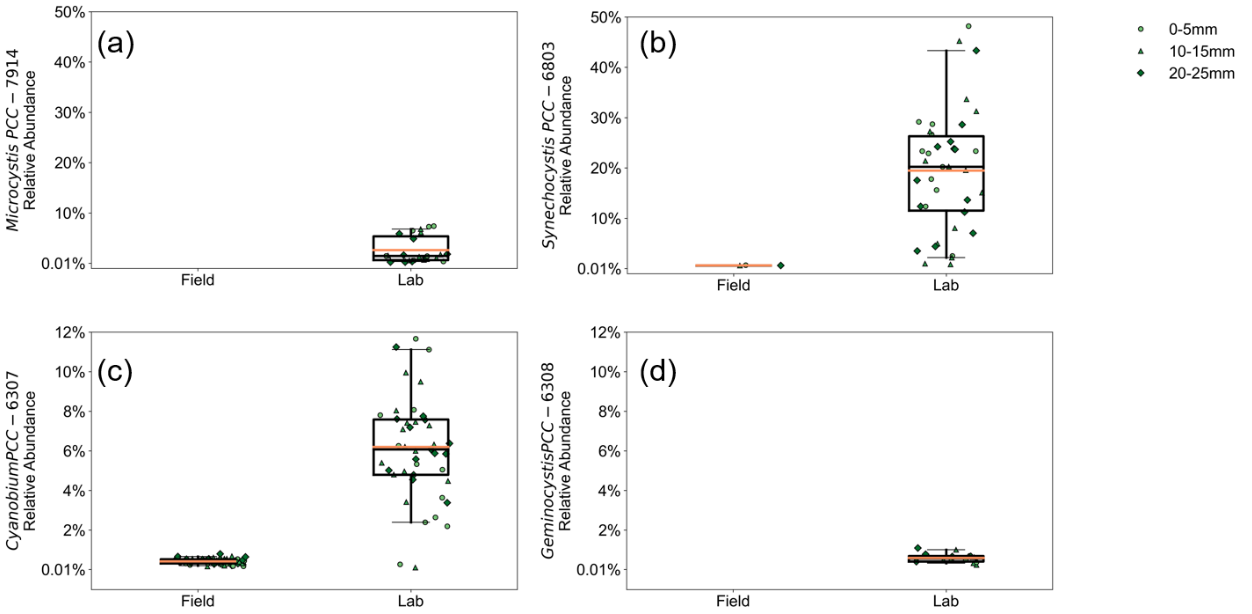

Figure S8. Relative abundance of four genera in field and lab bioreactor biomat samples. Points indicate specific genus clusters in each sample with different shapes indicating different depths: (a) *Microcysis PCC-7914*; (b) *Synechocysis PCC-6803*; (c) *Cyanobium PCC-6307*; (d) *Geminocystis PCC-6308*. The black line in the boxplot indicates the median value and the orange line indicates the average of all samples in that category.

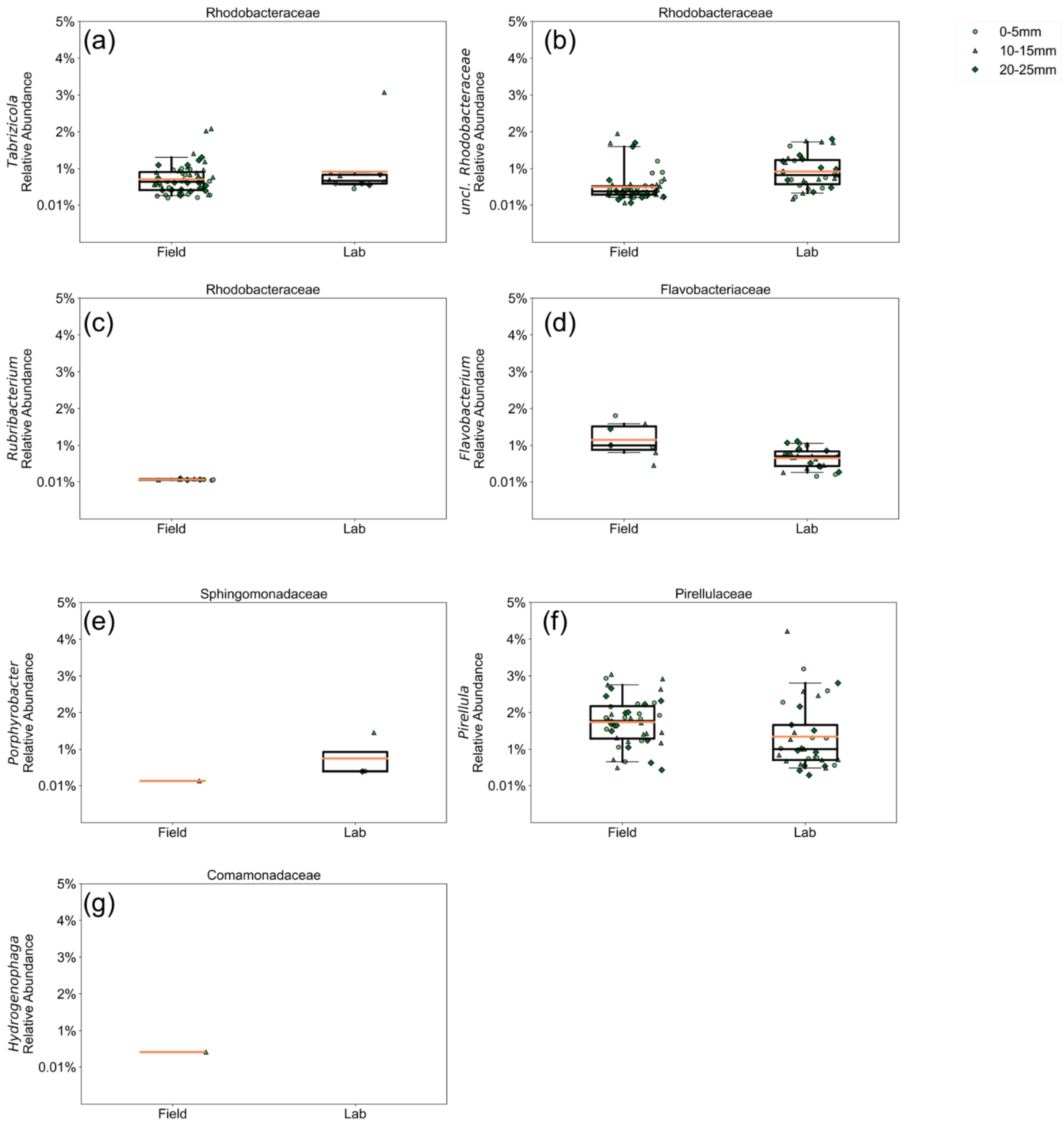

Figure S9. Relative abundance of putative phototrophs identified in the field and lab bioreactor biomat samples. Points indicate specific genus clusters in each sample with different shapes indicating different depths. The black line in the boxplot indicates the median value and the orange line indicates the average of all samples in that category.

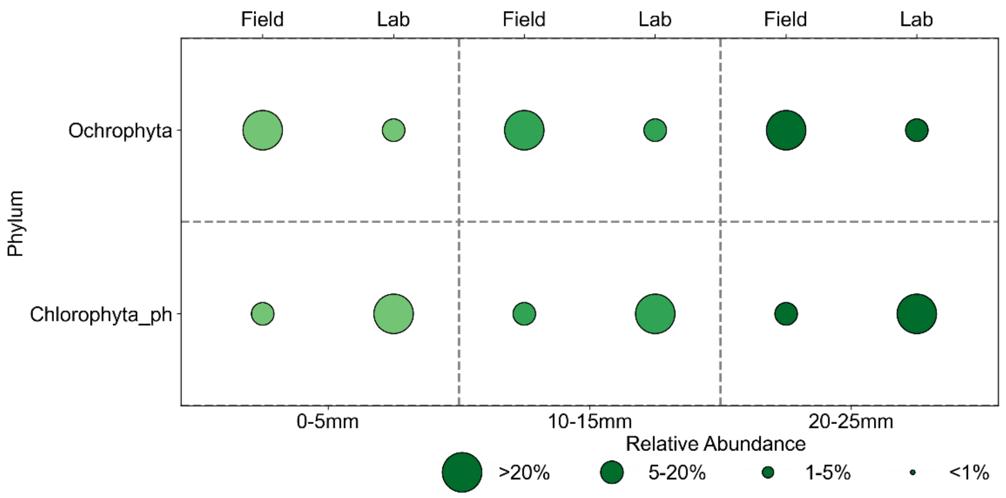

Figure S10. Relative abundance of Phylum Ochrophyta and Chlorophyta_ph for three depth sections by 18S rRNA gene sequencing. Marker sizes indicate the percentages of relative abundance that are the average values of all samples in that category, and the y-axis denotes the phylum level taxonomy.

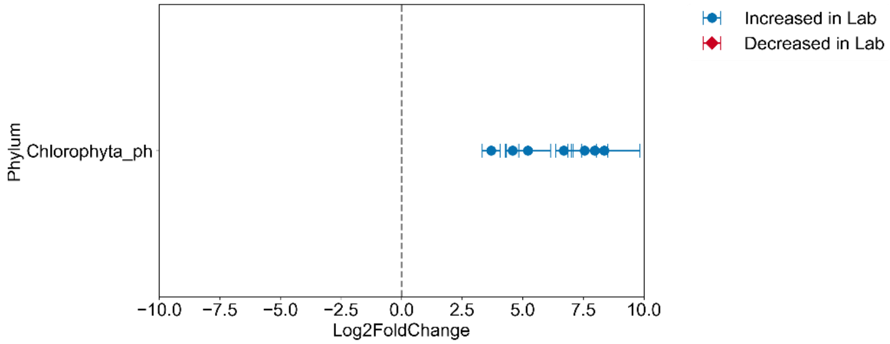

Figure S11. Differential abundance analysis between field and lab bioreactor biomat 18S rRNA samples for Phylum Ochrophyta and Chlorophyta_ph. Only |Log2FoldChange| > 4 and significant differences ($p<0.05$) were shown. The y-axis denotes the phylum level taxa. Blue circles indicate that the taxa were more enriched in lab bioreactor samples than in field samples.

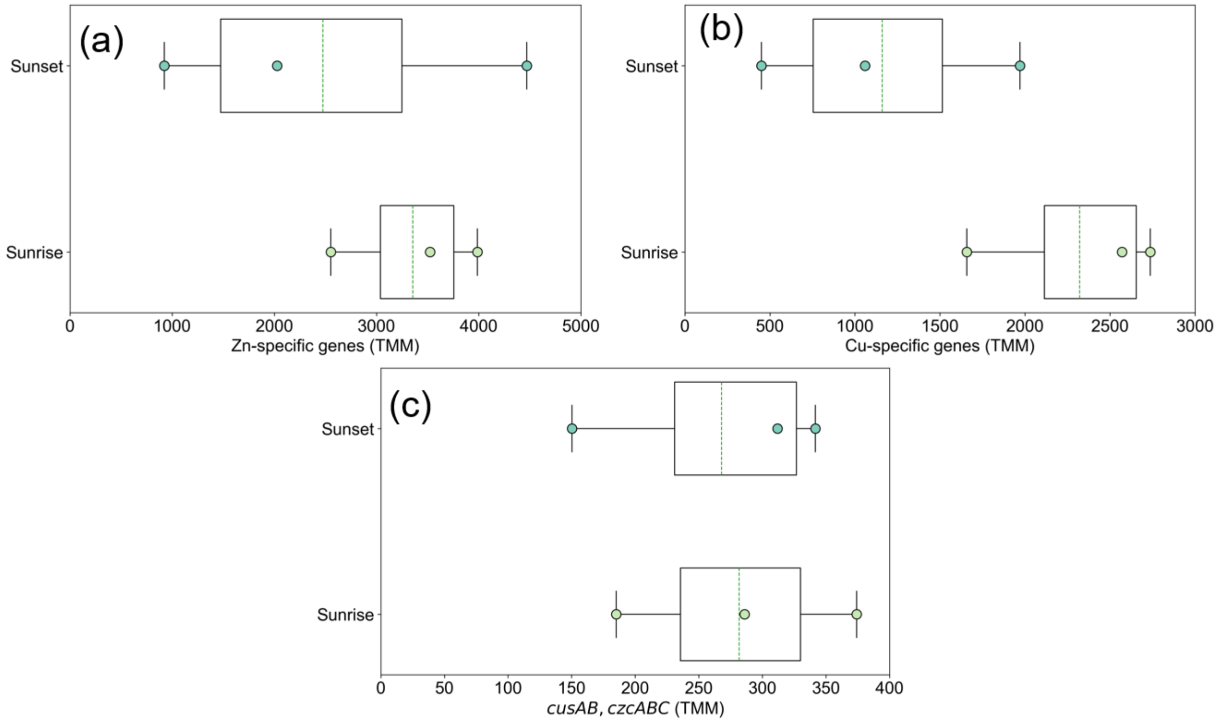

Figure S12. Transcripts encoding for zinc-specific processes (a), copper-specific processes (b), and divalent metal resistance (*cusAB*, *czcABC*) (c) from field biomat cores collected at sunset and sunrise in 2018. Criteria for searching these processes are described in detail in text and Table S16. Points here the sum of all transcripts from all MAGs within each category listed on the x-axis, for each 0 -5 mm core subsection (n=6). The dashed lines indicate the mean values.

Section S3. Methods for other aqueous analytes, characterization of organic matter, and results

For anions of research interest (nitrate, chloride, phosphate, and sulfate), samples were filtered through a 0.2µm PES filter and analyzed by ion chromatography (Thermo Fisher Scientific Dionex ICS-900). Total organic carbon (TOC) and total nitrogen (TN) samples were transferred into glass vials, acidified, and kept at 4°C prior to analyses. Dissolved organic carbon (DOC) samples were first filtered through a 0.45µm glass fiber filter before preserving in the same way as TOC samples. TOC, DOC, and TN were analyzed by Shimadzu TOC-550A Total Organic Carbon analyzer.

Sum of bicarbonate and carbonate concentrations in Table S7 were estimated based on calculations of the charge balance of the concentrations of all cations and anions in solution, then the relative bicarbonate to carbonate distribution was calculated based on pH and pKa1, pKa2 of carbonic acid. Cations in the calculations included H^+, Zn^{2+}, Cu^{2+}, Pb^{2+}, Ni^{2+} , Mn^{2+} , Al^{3+} , Ba^{2+} , Ca^{2+} , Fe^{3+} , K^+ , Mg^{2+} , Na^+ , and anions in the calculations included OH^-, Cl^-, NO_3^-, SO_4^{2-}, $H_3SiO_4^-$ (all data listed in Table S4, S6, S7). Alkalinity was then calculated based on Alk (mg/L as $CaCO_3$) = ($[HCO_3^-]$+ 2*$[CO_3^{2-}]$ + $[OH^-]$ - $[H^+]$) *100.

Aliquots of some DOC samples were analyzed for UV254 using a DU 800 spectrophotometer. Specific ultraviolet absorbance ($SUVA_{254}$) was calculated by normalizing the UV254 values with the respective DOC concentrations in mg/L following USEPA Method 415.3. Another aliquot of the DOC samples was used for excitation-emission matrix (EEM) fluorescence spectroscopy using a Horiba Aqualog 3D fluorometer and Aqualog 3.6 software. The experimental settings were conducted at an integration time of 1 s, excitation increments of 3 nm, and emission increments of 2.33 pixels. All EEM results were blank subtracted and normalized to 1 mg/L C from the DOC results. Five areas were divided according to previous studies,[15, 16] regions I and II are related to simple aromatic proteins; region III is related to fulvic acid-like organics; region IV is related to soluble microbial byproduct-like material; region V is related to humic acid-like organics. Humification index (HIX)[17], biological index (BIX)[18], and fluorescence index (FI)[19] were calculated as below using the fluorescence intensities obtained by EEM fluorescence spectroscopy:

$$HIX = \frac{\text{Sum of fluorescence intensities in 300 to 345 nm region}}{\text{Sum of fluorescence intensities in (300 to 345 nm region+435 to 480 nm region)}}\ at\ excitation\ 254\ nm \quad \text{(Eq.S2)}$$

$$BIX = \frac{\text{Fluorescence intensities at 380 nm}}{\text{Fluorescence intensities at 430 nm}}\ at\ excitation\ 310\ nm \quad \text{(Eq.S3)}$$

$$FI = \frac{\text{Fluorescence intensities at 470 nm}}{\text{Fluorescence intensities at 520 nm}}\ at\ excitation\ 370\ nm \quad \text{(Eq.S4)}$$

To explore the correlation between DOC concentrations and Zn/Cu removals, a set of batch microcosms with biomat were established with one subset exposed to light and the other subset completely kept in dark. Initial DOC concentrations were varied by generating biomat-derived DOC (10.4-12.1 mg/L) grown from flow-through bioreactor biomat (1.3-3.5 mg dry mass/ml) in separate flasks and then adding the biomat-derived DOC to the water column of the batch microcosms.

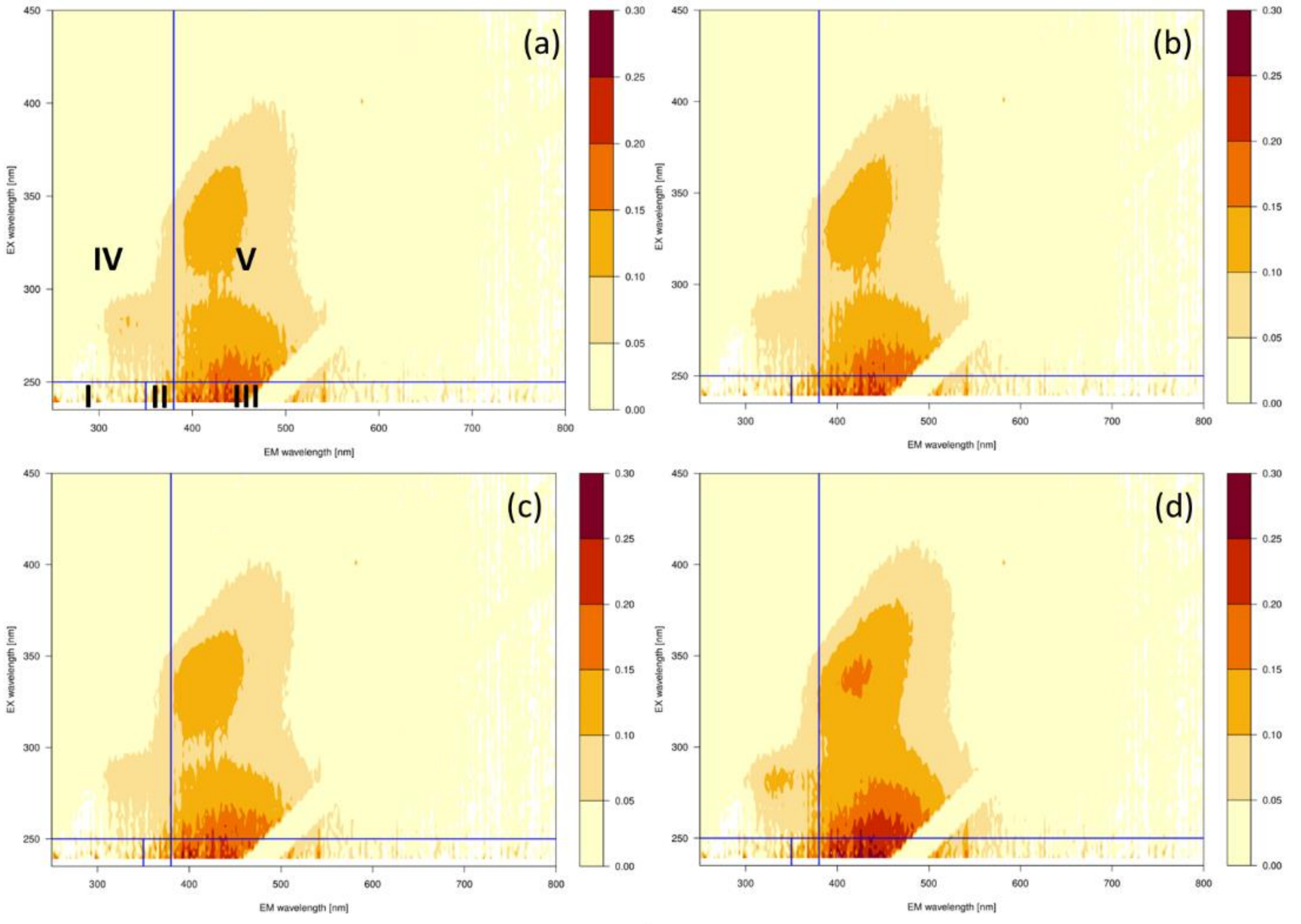

Figure S13. EEM fluorescence spectroscopy of DOC sampled at different time points (a, b, c, d) in the bioreactor after blank subtraction and normalization to 1 mg/L C.

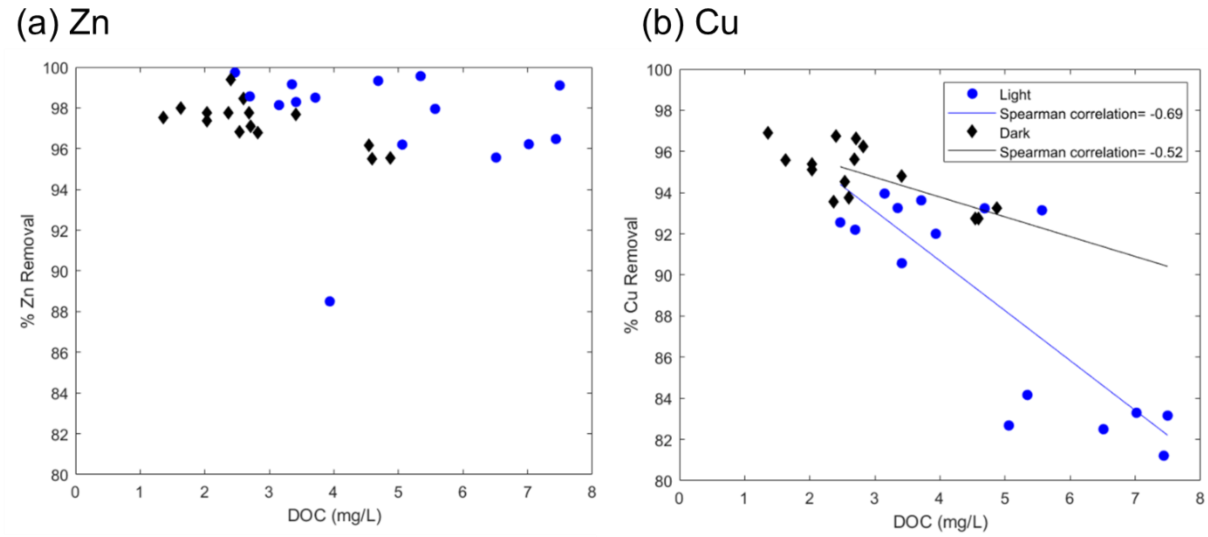

Figure S14. Correlations between DOC concentrations and removals of Zn (a) and Cu (b). Blue points indicate batch microcosms exposed to light. Grey diamonds indicate batch microcosms completely kept in dark. For all samples in (b), Spearman correlation coefficient = -0.72, $p<0.001$.

Section S4. Supplementary information for depth changes of metal phases

A modified sequential extraction protocol was used (Table S3) to explore possible phases of metals in the biomat formed in response to different experimental conditions. Four phases were operationally defined as water extractable; adsorbed/carbonates/exchangeable + labile organics (ACE+LO); oxidizable; and acid extractable. For biomat samples, 3 ml of deionized water was added, mixed, and centrifuged at 3000 g for 5 min, with the 'water extractable' fraction defined as the supernatant decanted into a new 15 ml tube. The remaining solid was added to 5 ml of 0.1M sodium pyrophosphate to extract the ACE+LO phase,[20] mixed on a shaker platform at 80 rpm for 1 h, centrifuged at 3000 g for 5 min, and the supernatant was decanted into a new tube. These two steps were repeated, and then the biomat was washed with 1 ml of deionized water twice, all supernatants all decanted and combined in the prior supernatant tube. For the oxidizable phase, the procedure was modified from that used by Tessier et al.[21] and used 30%

H_2O_2, mixed at 80 rpm for 5 h, then 2 ml of 3.2M NH_4Ac was added, mixed at 80 rpm for 30 min, and then the tubes were left open in the air to react and settle in a fume hood overnight. The mixture was centrifuged at 3000 g for 5 minutes, and the supernatant was decanted into a new 15 ml tube. The biomat was then washed with 0.5 ml of deionized water twice; all supernatants were combined with the prior 15 ml tube. For the final step, an acid extraction was performed according to the EPA Total Recoverable Method, EPA 200.2,[22] modified by decreasing both sample mass and acid volumes by a factor of 10.

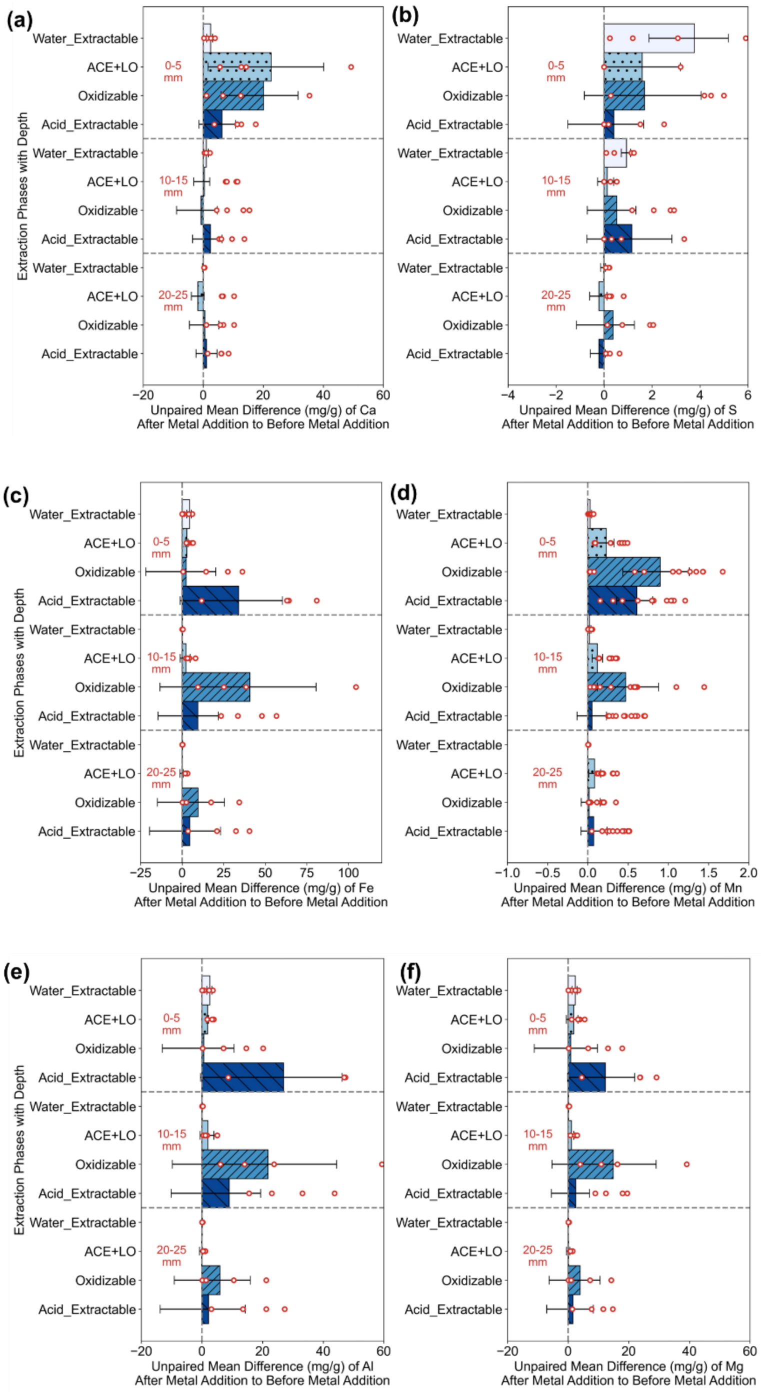

Figure S15. Depth profiles (mg/g) of major elements in the biomat: (a) Ca; (b) S; (c) Fe; (d) Mn; (e) Al; (f) Mg. Each bar shows the unpaired differences in the bioreactor biomat after metal addition compared to before metal addition for each sequential extraction phase - water extractable, adsorbed/carbonates/exchangeable + labile organics (A/C/E+LO), oxidizable, acid extractable - at each depth (0-5 mm, 10-15 mm, 20-25 mm). Red points represent the raw data included in the analysis (n=4 for each row for all elements except for Mn, n=12). The error bars are 95% confidence intervals after 5000 bootstrapping.

Section S5. Supplementary information for batch isotope microcosms

S5.1 Experimental set-ups

Batch microcosms (with biomat and bioreactor water sampled from the bioreactors in October 2022) were set up as 50 ml serum bottles, each with a rubber stopper on top connected to a needle with a 0.2µm polyethersulfone (PES) filter. The microcosms that were to be held in the dark were completely wrapped with electrical tape, whereas the rest of the microcosms were wrapped only at the bottom. For no-biomat control microcosms (solution only), all the sampling during the experiments was conducted under a nitrogen gas stream to avoid pH changes due to atmospheric carbon dioxide. All microcosms were placed under light with gentle shaking at 30 rpm. Enriched isotope solutions were diluted from isotopic stocks (Trace Sciences International Corp.) before amending to the microcosms to make a final mixture of 16.6 ± 1.1 µg dissolved ^{68}Zn and 8.8 ± 0.6 µg dissolved ^{65}Cu. The non-enriched isotope microcosms were amended with a solution mixture of 13.5 ± 1.3 µg dissolved Zn and 7.2 ± 0.7 µg dissolved Cu made directly from Zn and Cu chloride salt (Sigma-Aldrich). At each time point, water samples were taken through a needle and a 30 ml plastic syringe, and subsequently filtered through a 0.2µm PES filter (VWR). At the end of the experiments, overlaying supernatant was sampled for subsequent analyses described in Analytical Methods section. The remainder of the biomat was separated into two aliquots, one was used for direct acid extraction, and the other was used for sequential chemical extractions as described in S4.

S5.2 Analytical methods for isotope detection and correction

Metals of interest in the samples were analyzed by quadruple Inductively Coupled Plasma Mass Spectrometry (ICP-MS). For analytical quality checks, most of the elements were measured with their isotopes (except Br and Mn), including Zn-66, Zn-68, Cu-63, Cu-65, Pb-

208, Pb-207, Pb-206, Cd-110, Cd-112, Ni-60, Ni-62. Quality controls were performed by an in-line mixing of an internal standard (~10 ppb Indium) for instrumental drift corrections, blanks every 6-10 samples, external check standards every 20-30 samples, and calibration standards every 30-40 samples.

For the ACE+LO phase in sequential extractions, the extractant (*i.e.,* 0.1M sodium pyrophosphate) produced a high sodium background and led to interferences of $ArNa^+$ with ^{63}Cu on ICP-MS.[23] Thus, corrections were made to obtain accurate ^{63}Cu values. A calibration curve of four different sodium concentrations was made from dilutions of the 0.1M sodium pyrophosphate extractant solution, and the sodium concentrations in these calibration standards as well as samples were measured by ICP-AES. A linear relationship (R^2=0.9998) was obtained between the concentrations of sodium and ^{63}Cu interference. Interferences were back-calculated using the slope (0.0381) of the calibration curve and subtracted from the ^{63}Cu concentrations. In all other cases, background interferences from the sample matrix were minimal.

S5.3 Calculations for isotopic ratio enrichment and accumulation

The following equations calculate the accumulation of Zn in the biomat under the assumption that enriched isotopes accumulate in the biomat with minimal exchange with the non-enriched isotopes:[24]

$$Zn_{accum,phase\ i} = \frac{Zn_{tot,phase\ i}\left(f_{68-nat} - R_{final,phase\ i} \times f_{66-nat}\right)}{R_{final,phase\ i}\left(f_{66-enr} - f_{66-nat}\right) + f_{68-nat} - f_{68-enr}} \qquad (Eq. S5)$$

$$Fraction\ of\ Zn_{accum,phase\ i} = \frac{Zn_{accum,phase\ i}}{Zn_{accum,tot}} \qquad (Eq. S6)$$

where $Zn_{accum,phase\ i}$ is accumulated Zn in each extraction phase of the biomat at the end of the experiments (µg/g), $Zn_{tot,phase\ i}$ is total Zn in each extraction phase of the biomat at the end of

the experiments (μg/g), $R_{final,phase\ i}$ is the final $^{68}Zn/^{66}Zn$ ratio in each extraction phase of the biomat, f_{x-nat} is the fraction of a given isotope in the control biomat, f_{x-enr} is the fraction of a given isotope in the water column at the beginning of the experiments, $Zn_{accum,tot}$ is the total accumulated Zn in the biomat at the end of the experiments (μg/g). Cu accumulation was calculated in the same way with ^{65}Cu replacing ^{68}Zn and ^{63}Cu replacing ^{66}Zn in Eq.S5 and S6.

S5.4 Supplementary results for batch isotope microcosms

DOC characterization (Figure S16) was conducted for selected groups at the end of the experiments using the method described in SI Section S3. To explore the relationship between metals and other major elements of each extraction phase, Spearman correlation analysis was performed in MATLAB ver. R2020b (Figure S17).[25]

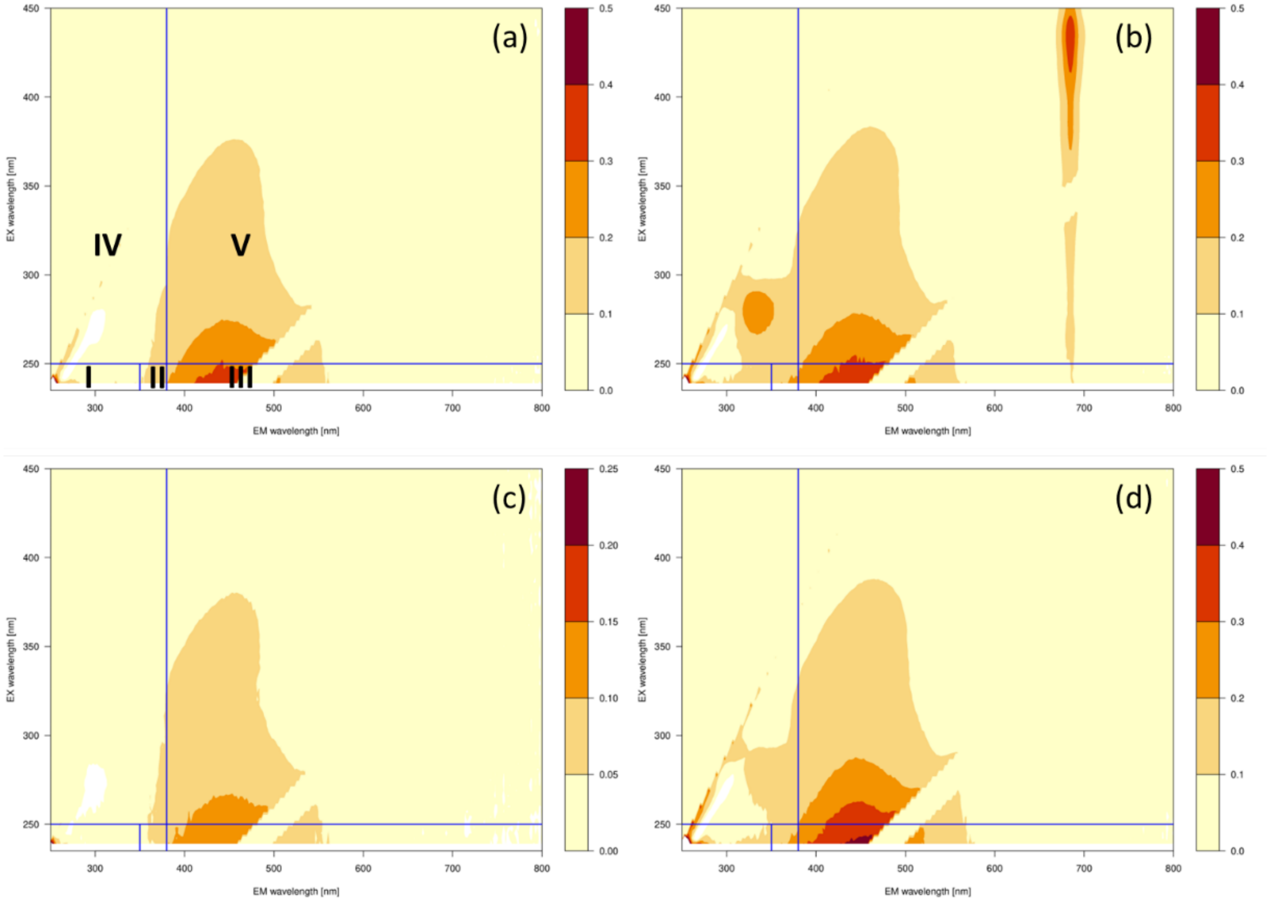

Figure S16. EEM fluorescence spectroscopy of DOC sampled at the end of the batch microcosm experiments for different groups (a: unenriched diel biomat microcosms, b: enriched diel biomat microcosms, c: enriched dark biomat microcosms, d: no-addition diel biomat microcosms) after blank subtraction and normalization to 1 mg/L C. Corresponding data are shown in Table S13.

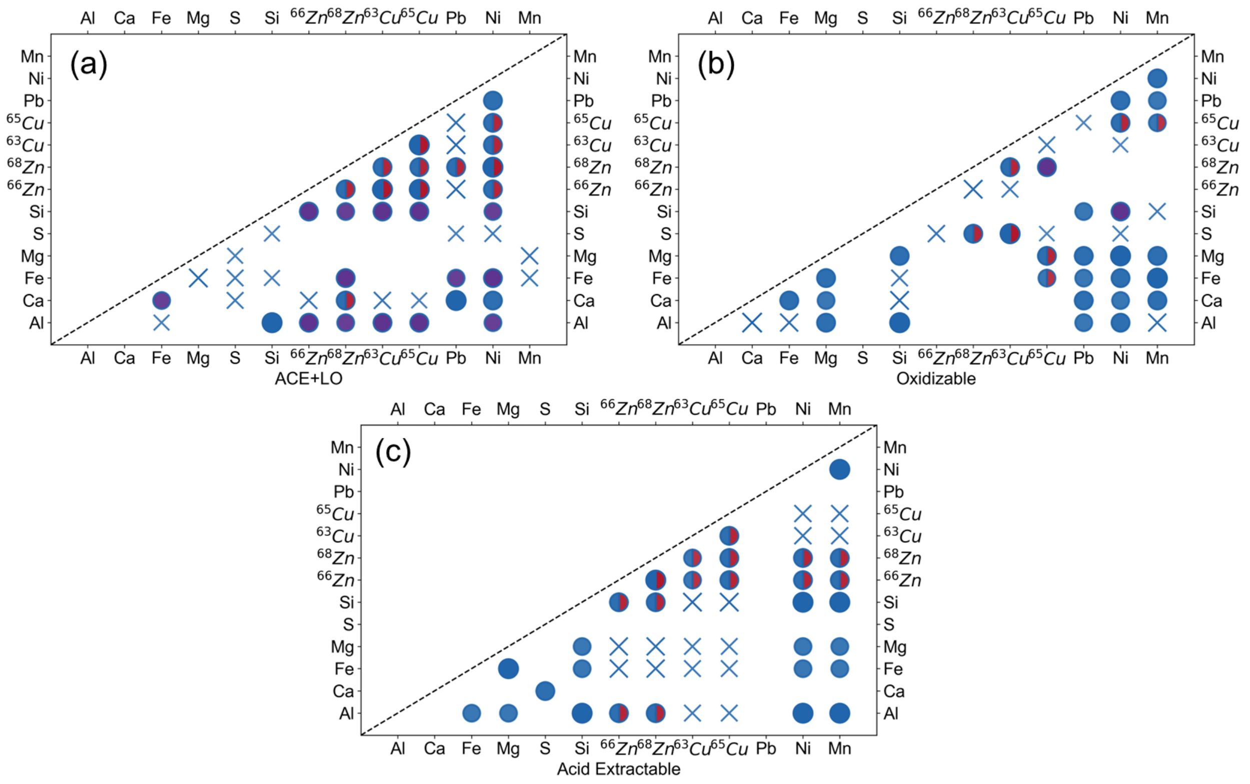

Figure S17. Spearman correlations of metals and major elements in each extraction phase: (a) ACE+LO; (b) Oxidizable; (c) Acid extractable. Blue-red points and blue-only points represent significant correlations between the two elements (Spearman correlation coefficients >0.7, $p<0.05$) in all groups (n=12). Purple points represent significant correlations between the two elements in isotopic enriched groups (n=6) but not the non-enriched groups (n=6). Blue crosses represent significant correlations between the two elements in non-enriched groups (n=6) but not the enriched groups (n=6). Blank means that there are no significant correlations in any group.

Section S6. PHREEQC models for diel changes

A PHREEQC[26] model was set up to simulate diel variations of dissolved zinc and copper. The conceptual setup of the model is shown in Figure S18. In brief, we modified diel variations of carbon dioxide to condition the model system pHs to match the pHs we observed in the experiments. With that and bioreactor water chemistry data and dissolved oxygen concentrations in the experiments as the model inputs, the model generated dissolved zinc/copper concentration

changes as outputs (Figure 5b and 5c), in which several chemical processes were involved. These processes were used to infer the possible mechanisms that caused similar changes in the dissolved zinc/copper diel with the caveat that these need to be interpreted with the experimental results to have a more solid confirmation of the mechanisms.

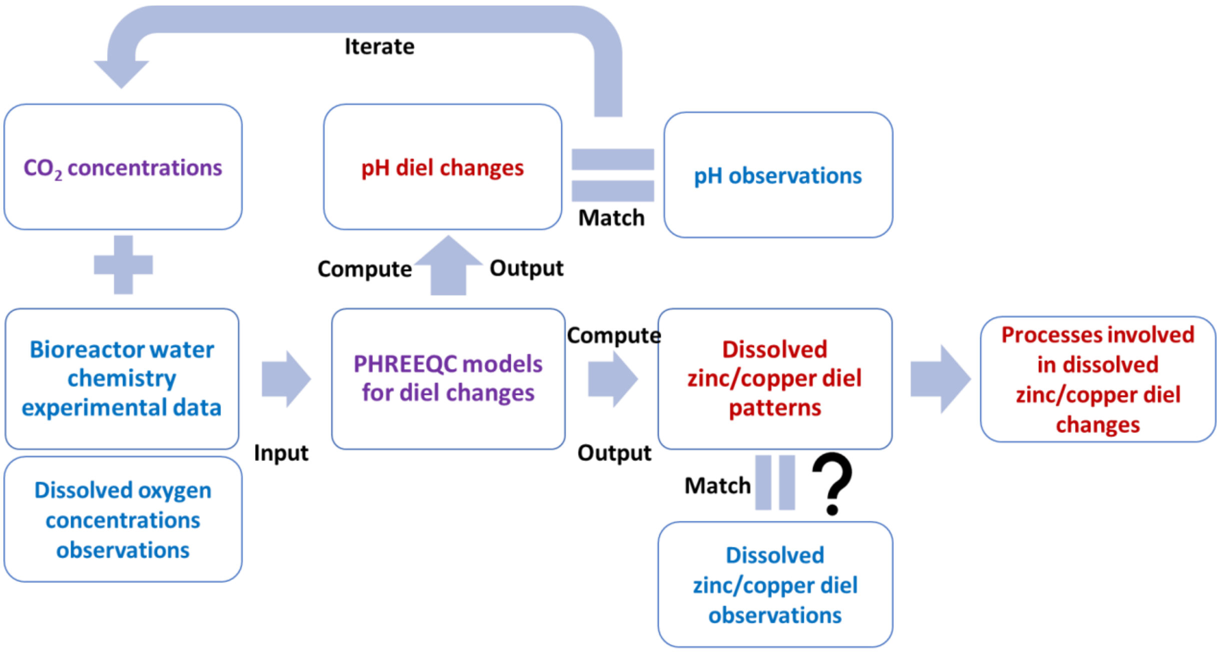

Figure S18. Conceptual setup of the PHREEQC model. Blue texts indicate model inputs from experimental data, purple texts indicate model inputs from manual setups, and red texts indicate model output predictions.

A previous model was established on biomat in a completely mixed system for metal sorption/surface complexation, and literature parameters of surface site densities from ferrihydrite-bacteria composites (strong and weak sites of ferrihydrite, bacteria carboxyl group, phosphoryl group, amine group) and diatoms (diatom carboxyl group, silanol group) were optimized to fit the results from biomat.[27] In addition to this model, we incorporated some other processes based on the bioreactor experiment data: zinc to calcite sorption;[28, 29] humic and fulvic

substance complexation with zinc and copper from WHAM and Model VI in Visual Minteq databases;[30] microbial exudate compounds that had existing complexation reaction constants in Visual Minteq databases were also added based on studies on *Synechocystis PCC 6803* and other photosynthetic organisms (Table S15).[31, 32]

References

1. Vega, M. A. P.; Scholes, R. C.; Brady, A. R.; Daly, R. A.; Narrowe, A. B.; Bosworth, L. B.; Wrighton, K. C.; Sedlak, D. L.; Sharp, J. O., Pharmaceutical Biotransformation is Influenced by Photosynthesis and Microbial Nitrogen Cycling in a Benthic Wetland Biomat. *Environ. Sci. Technol.* **2022**.
2. Jasper, J. T.; Jones, Z. L.; Sharp, J. O.; Sedlak, D. L., Nitrate Removal in Shallow, Open-Water Treatment Wetlands. *Environ. Sci. Technol.* **2014,** *48*, (19), 11512-11520.
3. Kadlec, R. H.; Wallace, S., *Treatment wetlands*. CRC press: 2008.
4. Schubert, M.; Lindgreen, S.; Orlando, L., AdapterRemoval v2: rapid adapter trimming, identification, and read merging. *BMC Research Notes* **2016,** *9*, (1), 88.
5. Callahan, B. J.; McMurdie, P. J.; Rosen, M. J.; Han, A. W.; Johnson, A. J.; Holmes, S. P., DADA2: High-resolution sample inference from Illumina amplicon data. *Nat Methods* **2016,** *13*, (7), 581-3.
6. Yilmaz, P.; Parfrey, L. W.; Yarza, P.; Gerken, J.; Pruesse, E.; Quast, C.; Schweer, T.; Peplies, J.; Ludwig, W.; Glöckner, F. O., The SILVA and "All-species Living Tree Project (LTP)" taxonomic frameworks. *Nucleic Acids Research* **2013,** *42*, (D1), D643-D648.
7. Bolyen, E.; Rideout, J. R.; Dillon, M. R.; Bokulich, N. A.; Abnet, C. C.; Al-Ghalith, G. A.; Alexander, H.; Alm, E. J.; Arumugam, M.; Asnicar, F.; Bai, Y.; Bisanz, J. E.; Bittinger, K.; Brejnrod, A.; Brislawn, C. J.; Brown, C. T.; Callahan, B. J.; Caraballo-Rodríguez, A. M.; Chase, J.; Cope, E. K.; Da Silva, R.; Diener, C.; Dorrestein, P. C.; Douglas, G. M.; Durall, D. M.; Duvallet, C.; Edwardson, C. F.; Ernst, M.; Estaki, M.; Fouquier, J.; Gauglitz, J. M.; Gibbons, S. M.; Gibson, D. L.; Gonzalez, A.; Gorlick, K.; Guo, J.; Hillmann, B.; Holmes, S.; Holste, H.; Huttenhower, C.; Huttley, G. A.; Janssen, S.; Jarmusch, A. K.; Jiang, L.; Kaehler, B. D.; Kang, K. B.; Keefe, C. R.; Keim, P.; Kelley, S. T.; Knights, D.; Koester, I.; Kosciolek, T.; Kreps, J.; Langille, M. G. I.; Lee, J.; Ley, R.; Liu, Y.-X.; Loftfield, E.; Lozupone, C.; Maher, M.; Marotz, C.; Martin, B. D.; McDonald, D.; McIver, L. J.; Melnik, A. V.; Metcalf, J. L.; Morgan, S. C.; Morton, J. T.; Naimey, A. T.; Navas-Molina, J. A.; Nothias, L. F.; Orchanian, S. B.; Pearson, T.; Peoples, S. L.; Petras, D.; Preuss, M. L.; Pruesse, E.; Rasmussen, L. B.; Rivers, A.; Robeson, M. S.; Rosenthal, P.; Segata, N.; Shaffer, M.; Shiffer, A.; Sinha, R.; Song, S. J.; Spear, J. R.; Swafford, A. D.; Thompson, L. R.; Torres, P. J.; Trinh, P.; Tripathi, A.; Turnbaugh, P. J.; Ul-Hasan, S.; van der Hooft, J. J. J.; Vargas, F.; Vázquez-Baeza, Y.; Vogtmann, E.; von Hippel, M.; Walters, W.; Wan, Y.; Wang, M.; Warren, J.; Weber, K. C.; Williamson, C. H. D.; Willis, A. D.; Xu, Z. Z.; Zaneveld, J. R.; Zhang, Y.; Zhu, Q.; Knight, R.; Caporaso, J. G., Reproducible, interactive, scalable and extensible microbiome data science using QIIME 2. *Nature Biotechnology* **2019,** *37*, (8), 852-857.

8. McMurdie, P. J.; Holmes, S., phyloseq: An R Package for Reproducible Interactive Analysis and Graphics of Microbiome Census Data. *PLoS One* **2013,** *8*, (4), e61217.
9. Andersen, K. S.; Kirkegaard, R. H.; Karst, S. M.; Albertsen, M., ampvis2: an R package to analyse and visualise 16S rRNA amplicon data. *bioRxiv* **2018**, 299537.
10. Love, M. I.; Huber, W.; Anders, S., Moderated estimation of fold change and dispersion for RNA-seq data with DESeq2. *Genome Biol.* **2014,** *15*, (12), 550.
11. Gu, Z.; Eils, R.; Schlesner, M., Complex heatmaps reveal patterns and correlations in multidimensional genomic data. *Bioinformatics* **2016,** *32*, (18), 2847-2849.
12. Leo Lahti; Sudarshan Shetty; et, a. l., Tools for microbiome analysis in R. Version. **2017**.
13. Oksanen, J.; Simpson, G. L.; Blanchet, F. G.; Kindt, R.; Legendre, P.; Minchin, P. R.; O'Hara, R. B.; Solymos, P.; Stevens, M. H. H.; Szoecs, E.; Wagner, H.; Barbour, M.; Bedward, M.; Bolker, B.; Borcard, D.; Carvalho, G.; Chirico, M.; Caceres, M. D.; Durand, S.; Evangelista, H. B. A.; FitzJohn, R.; Friendly, M.; Furneaux, B.; Hannigan, G.; Hill, M. O.; Lahti, L.; McGlinn, D.; Ouellette, M.-H.; Cunha, E. R.; Smith, T.; Stier, A.; Braak, C. J. F. T.; Weedon, J., vegan: Community Ecology Package. R package version 2.6-2. **2022**.
14. Vega, M. A. P.; Scholes, R. C.; Brady, A. R.; Daly, R. A.; Narrowe, A. B.; Vanzin, G. F.; Wrighton, K. C.; Sedlak, D. L.; Sharp, J. O., Methane-Oxidizing Activity Enhances Sulfamethoxazole Biotransformation in a Benthic Constructed Wetland Biomat. *Environ. Sci. Technol.* **2023**.
15. Chen, W.; Westerhoff, P.; Leenheer, J. A.; Booksh, K., Fluorescence Excitation−Emission Matrix Regional Integration to Quantify Spectra for Dissolved Organic Matter. *Environ. Sci. Technol.* **2003,** *37*, (24), 5701-5710.
16. Leonard, L. T.; Vanzin, G. F.; Garayburu-Caruso, V. A.; Lau, S. S.; Beutler, C. A.; Newman, A. W.; Mitch, W. A.; Stegen, J. C.; Williams, K. H.; Sharp, J. O., Disinfection byproducts formed during drinking water treatment reveal an export control point for dissolved organic matter in a subalpine headwater stream. *Water Res. X* **2022,** *15*, 100144.
17. Ohno, T., Fluorescence Inner-Filtering Correction for Determining the Humification Index of Dissolved Organic Matter. *Environ. Sci. Technol.* **2002,** *36*, (4), 742-746.
18. Huguet, A.; Vacher, L.; Relexans, S.; Saubusse, S.; Froidefond, J. M.; Parlanti, E., Properties of fluorescent dissolved organic matter in the Gironde Estuary. *Org. Geochem.* **2009,** *40*, (6), 706-719.
19. Cory, R. M.; McKnight, D. M., Fluorescence Spectroscopy Reveals Ubiquitous Presence of Oxidized and Reduced Quinones in Dissolved Organic Matter. *Environ. Sci. Technol.* **2005,** *39*, (21), 8142-8149.
20. Carlsson, E.; Thunberg, J.; Öhlander, B.; Holmström, H., Sequential extraction of sulfide-rich tailings remediated by the application of till cover, Kristineberg mine, northern Sweden. *Sci. Total Environ.* **2002,** *299*, (1), 207-226.
21. Tessier, A.; Campbell, P. G. C.; Bisson, M., Sequential extraction procedure for the speciation of particulate trace metals. *Anal. Chem.* **1979,** *51*, (7), 844-851.
22. U.S. Environmental Protection Agency. *Method 200.2, Revision 2.8: Sample Preparation Procedure for Spectrochemical Determination of Total Recoverable Elements* **1994**.
23. May, T. W.; Wiedmeyer, R. H., A table of polyatomic interferences in ICP-MS. *Atom. Spectrosc.* **1998,** *19*, (5), 150-155.
24. Wolf, R. E.; Todd, A. S.; Brinkman, S.; Lamothe, P. J.; Smith, K. S.; Ranville, J. F., Measurement of total Zn and Zn isotope ratios by quadrupole ICP-MS for evaluation of Zn

uptake in gills of brown trout (Salmo trutta) and rainbow trout (Oncorhynchus mykiss). *Talanta* **2009,** *80*, (2), 676-684.

25. Trauth, M.; Sillmann, E.; Gebbers, R., *MATLAB® Recipes for Earth Sciences*. 4 ed.; Springer Berlin Heidelberg: 2015.

26. U.S. Geological Survey. *PHREEQC version 3 (https://www.usgs.gov/software/phreeqc-version-3, Retrieved January 13th, 2021)* **2021**.

27. Yang, Z.; Acker, S. M.; Brady, A. R.; Rodríguez, A. A.; Paredes, L. M.; Ticona, J.; Mariscal, G. R.; Vanzin, G. F.; Ranville, J. F.; Sharp, J. O., Heavy metal removal by the photosynthetic microbial biomat found within shallow unit process open water constructed wetlands. *Sci. Total Environ.* **2023,** *876*, 162478.

28. Zachara, J. M.; Cowan, C. E.; Resch, C. T., Sorption of divalent metals on calcite. *Geochim. Cosmochim. Acta* **1991,** *55*, (6), 1549-1562.

29. Duan, Y. R.; He, H. T.; Liu, W. C.; Gou, W. X.; Wang, Z.; Liu, P.; Zhang, J.; Peacock, C. L.; Li, W., Coupling stable isotope analyses and X-ray absorption spectroscopy to investigate the molecular mechanism of zinc sorption by calcite. *Geochim. Cosmochim. Acta* **2025,** *390*, 232-250.

30. Lofts, S.; Tipping, E., Solid-solution metal partitioning in the Humber rivers: application of WHAM and SCAMP. *Sci. Total Environ.* **2000,** *251*, 381-399.

31. Pereira, S.; Zille, A.; Micheletti, E.; Moradas-Ferreira, P.; De Philippis, R.; Tamagnini, P., Complexity of cyanobacterial exopolysaccharides: composition, structures, inducing factors and putative genes involved in their biosynthesis and assembly. *Fems Microbiol. Rev.* **2009,** *33*, (5), 917-941.

32. Haavisto, V.; Landry, Z.; Pontrelli, S., High-throughput profiling of metabolic responses to exogenous nutrients in <i>Synechocystis</i> sp. PCC 6803. *mSystems* **2024,** *9*, (4), e00227-24.
